# Perinatal maternal depression and the risk of childhood asthma in offspring: A meta-analysis

**DOI:** 10.1371/journal.pone.0310647

**Published:** 2024-09-30

**Authors:** Xiqun Jia, Liang Lu, Shiyang Lou, Siyu Han, Linli Deng, Shuhua Liu

**Affiliations:** 1 Department of Pediatrics, Shenzhen Longhua District Central Hospital, Shenzhen, China; 2 Department of Neonatal, Shenzhen Longhua District Central Hospital, Shenzhen, China; Chiba Daigaku, JAPAN

## Abstract

**Background:**

Previous studies have yielded conflicting results regarding the link between maternal perinatal depression and asthma in children. To provide a clearer understanding of this relationship, a comprehensive meta-analysis was carried out to evaluate the association mentioned above.

**Methods:**

A comprehensive review of observational studies was conducted by searching electronic databases including Medline, Embase, and Web of Science. The data were combined using a randomized-effects model taking into account potential variations. Subgroup analyses were performed to evaluate the possible impact of study characteristics on outcomes.

**Results:**

Ten cohort studies, which included 833,230 mother-child pairs, were examined in the analysis. Maternal depressive symptoms during the perinatal period were associated with an increased risk of asthma in offspring (risk ratio [RR]: 1.24, 95% confidence interval [CI]: 1.19 to 1.30, *p* < 0.001; I^2^ = 0%). Further sensitivity analyses restricted to multivariate studies (RR: 1.24, 95% CI: 1.19 to 1.30, *p* < 0.001; I^2^ = 0%) or studies where asthma was diagnosed in children aged three years or older (RR: 1.24, 95% CI: 1.19 to 1.30, *p* < 0.001; I^2^ = 0%) revealed consistent outcomes. Subgroup analyses according to study design, methods for the diagnosis of maternal depression, timing for the evaluation of maternal depression, methods for the validation of asthma in offspring, adjustment of maternal smoking during pregnancy and of maternal asthma, or study quality score showed similar results (*p* for subgroup difference all > 0.05).

**Conclusions:**

Maternal perinatal depression appears to be significantly linked to a higher occurrence of childhood asthma in children.

## Introduction

Asthma is a chronic respiratory condition that affects children and adults, characterized by episodes of wheezing, breathlessness, chest tightness, and coughing [1]. While asthma is commonly associated with allergic responses (atopic asthma), non-atopic asthma subgroups also exist and have diverse mechanisms, including initiation by infections and environmental factors [2]. Children with asthma encounter diverse breathing issues and restricted physical movement, leading to a notable decline in quality of life [3,4]. Moreover, instances of acute asthma attacks triggered by factors like respiratory infections have increasingly become a primary reason for emergency room visits or hospital admissions [5,6]. As such, it is crucial to identify new risk factors for the development of asthma. It is becoming increasingly evident that the likelihood of developing asthma may be influenced during pregnancy or early childhood events [7,8]. A number of maternal factors during the perinatal period have been linked to an increased likelihood of children developing asthma, including smoking by mothers during pregnancy [9], maternal history of asthma [10], and maternal obesity before conception [11].

Both prenatal and postnatal maternal depression have been linked to an increased risk of asthma in offspring, though through potentially different mechanisms. Prenatal depression can impact fetal development directly through increased cortisol exposure and altered immune regulation [12]. In contrast, postnatal depression may influence the asthma risk of the children through mechanisms related to early-life stress and altered maternal-infant interactions. For example, postnatal depression could affect breastfeeding practices and exposure to environmental triggers, impacting the respiratory health of the children [13]. Therefore, examining both prenatal and postnatal depression is crucial to understanding the full scope of maternal depression’s impact on childhood asthma risk. On the other hand, prenatal depression can overstretch into the postnatal period, making it clinically difficult to distinguish exposures solely on prenatal or postnatal maternal depression. Accordingly, it is important to determine the influence of perinatal maternal depression on the risk of childhood asthma in offspring.

Several studies suggest that depression during the perinatal period in mothers is linked to a higher likelihood of asthma in their children [14–18], while in other studies, the association was not statistically significant [19–23]. Although several meta-analyses have been conducted, the significance of the association between maternal depression and asthma risk in offspring remains unclear. An initial meta-analysis from 2018, which pooled findings from eight studies, indicated that prenatal maternal exposure to various forms of stress was linked to an increased likelihood of asthma onset in offspring [24]. Although the authors separately analyzed the influences according to the type of stressor, only one reported a specific association between maternal prenatal depression and asthma risk in offspring [24]. Subsequently, a 2021 meta-analysis incorporating four studies also suggested that maternal prenatal stress might be connected to a heightened risk of asthma in offspring [25]. Nevertheless, due to the inclusion of studies with different types of maternal stress exposures (e.g., anxiety, depression), significant heterogeneity persisted within this analysis as well [25]. In addition, it remains unknown if the association between maternal depression and childhood asthma in offspring is significantly affected by study characteristics such as study design and methods for validating the diagnosis of both maternal depression and childhood asthma. Accordingly, this study conducted a meta-analysis to examine the potential link between maternal perinatal depression and an elevated risk of asthma in children, as well as to determine if this association holds true for both prenatal and postpartum depression.

## Methods

In conducting and reporting the meta-analysis, we adhered to the guidelines outlined in the Preferred Reporting Items for Systematic Reviews [26,27] and Meta-Analyses and Cochrane Handbook [28]. The protocol of the meta-analysis has been registered in PROSPERO with the registration code CRD42024545297. Institutional Review Board approval was not required because this is a meta-analysis.

### Database search

We conducted a thorough search of the Medline, Embase, Cochrane Library, and Web of Science electronic databases to find pertinent studies using the following specific terms: ("pregnant" OR "pregnancy" OR "antenatal" OR "antepartum" OR "perinatal" OR "prenatal" OR "postnatal" OR "postpartum" OR "postnatal" OR "ante natal" OR "ante partum") AND ("depression" OR "depressive" OR "mood" OR "affective disorder") AND ("asthma" OR "wheeze" OR "wheezing") AND ("child" OR "children" OR "adolescent" OR "pediatric" OOR "infant" OR "neonate" OR "newborn" OR "toddler"). The literature search was completed on January 31, 2024. Only studies that involved human subjects that were published in English in peer-reviewed journals were included. The terms related to “wheezing” were also included in the search strategy because diagnosing asthma in younger children (e.g. < 3 years) is often challenging [29]. For some studies, the authors may report the outcome of wheeze but also will have the information on the subsequent diagnosis of childhood asthma. To avoid missing of such studies, terms related to “wheezing” were also included in search strategy, but only studies that reported the incidence of childhood asthma were included in the meta-analysis. Additionally, we manually checked the reference lists of related original and review articles to potentially identify any original studies that were not included.

### Inclusion and exclusion criteria

We utilized the PICOS framework to establish the eligibility criteria.

P (Participants): Females and their children;

I (Intervention/exposure): Women experiencing symptoms of depression during the perinatal period following the birth of their child; this period spans from one year before to 18~24 months after delivery, as defined in previous literature [30].

C (Control/comparator): Women not exhibiting depressive symptoms during the perinatal period surrounding childbirth;

O (Outcome): Occurrence of asthma in children;

S (Study design): Cohort studies, encompassing both prospective and retrospective cohorts.

The diagnostic approaches for identifying depression and asthma were in line with the techniques utilized in the original publications. Excluded from consideration were reviews, editorials, meta-analyses, studies that did not specifically assess maternal depression during the perinatal period, or studies that did not report on the incidence of asthma in offspring.

### Study quality assessment and data extraction

The study quality was evaluated using the Newcastle–Ottawa Scale (NOS) [31], which comprised three key areas: definition of study groups, comparability between groups, and validation of outcomes. The NOS consists of nine criteria, with a score ranging from 1 to 9 stars based on meeting each criterion; a higher score indicating better study quality. Two researchers (LL and SL) independently conducted electronic database searches, data extraction, and assessment of study quality according to predefined inclusion criteria. Any discrepancies were resolved through discussion with the corresponding author. The extracted data included details about the studies (such as authors, countries, publication year, and design), numbers of mother-child pairs involved along with methods and timing for assessing maternal depression during pregnancy or postpartum period; children’s age at asthma diagnosis; offspring gender; methods used to confirm asthma diagnosis in children; number of children affected by asthma; as well as variables adjusted in regression analysis investigating the association between maternal perinatal depression and childhood asthma. Missing study or patient characteristics were labeled as "not reported" (NR) in the data extraction table, and studies without available outcome data were omitted from the meta-analysis. The final data extraction was performed on March 12, 2024 by LL and SL.

### Statistical analysis

Risk ratios (RRs) and their corresponding 95% confidence intervals (CIs) were chosen as the primary outcome measure to assess the link between maternal perinatal depression and asthma incidence in children. RRs and standard errors were computed from either 95% CIs or p values, with an additional log transformation conducted for variance stabilization and distribution normalization [28]. Statistical heterogeneity was assessed using the Cochrane Q test along with estimation of the I^2^ statistic, considering heterogeneity significant if I^2^ >50% [32]. In view of the potential clinical heterogeneity in timing of maternal depression of the included studies, as well as heterogeneity of methods for evaluating maternal depression and offspring asthma outcome, a random-effects model was used to pool the results even if the statistical heterogeneity was low [28]. Sensitivity analyses involved exclusion of one dataset at a time to evaluate result stability, focusing on studies with multivariate analyses and asthma diagnosed in children aged three years or older, because diagnosing asthma in younger children is often challenging [29]. Subgroup analysis explored associations between maternal perinatal depression and offspring’s asthma based on study design, methods for maternal depression diagnosis, timing of depression evaluation, validation methods for offspring’s asthma diagnosis, adjustment for maternal smoking during pregnancy, adjustment for maternal history of asthma, and study quality scores. Funnel plots were used alongside visual inspection of symmetry to reflect publication bias while Egger’s regression asymmetry test evaluated potential bias further [33]. Statistical software, including RevMan (Version 5.1; Cochrane Collaboration) and Stata (version 12.0; Stata Corporation), were used for these analyses.

## Results

### Study inclusion

**Fig 1:**
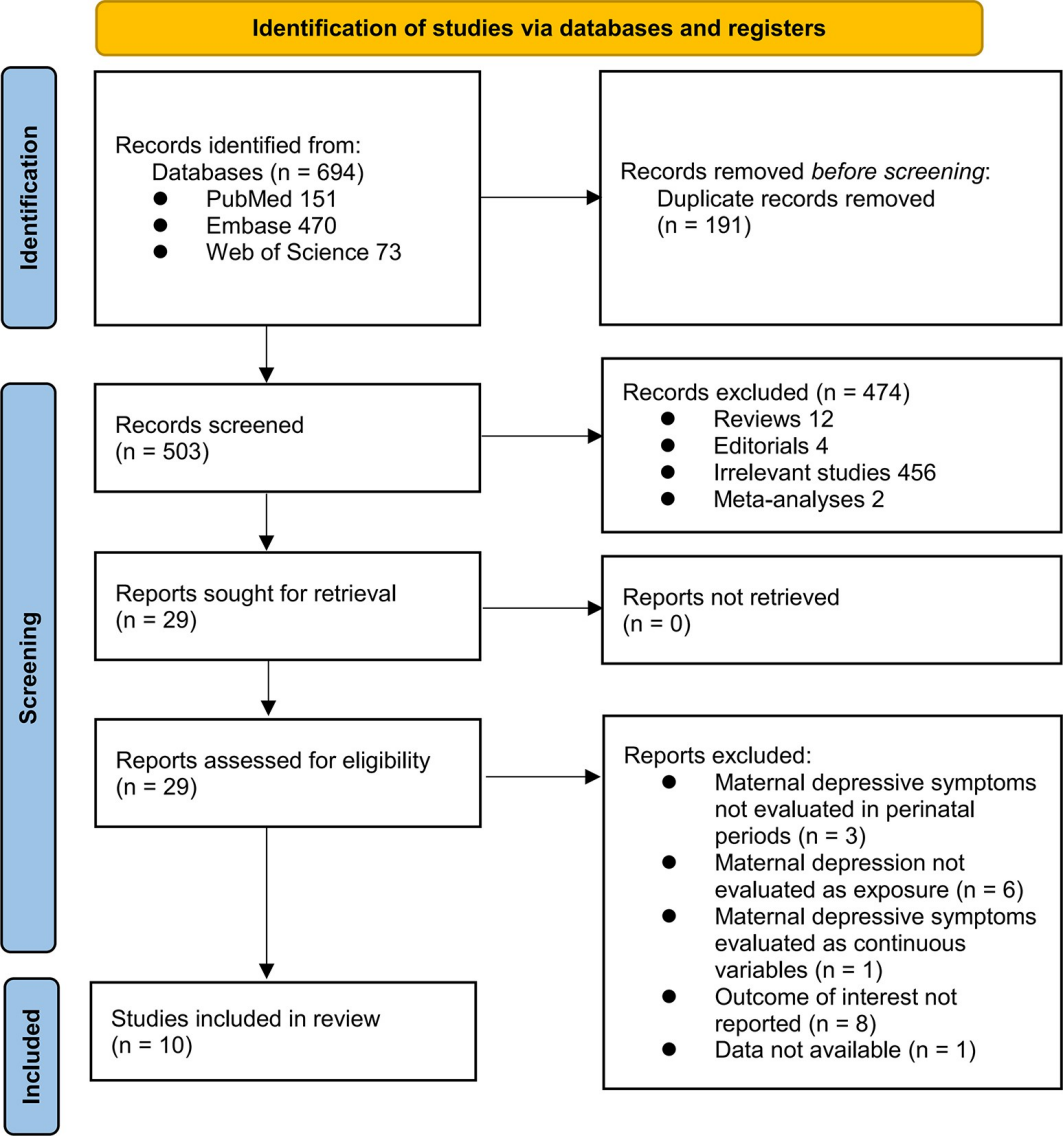
Flowchart of database search and study selection.

The process of selecting relevant studies for inclusion in the meta-analysis is depicted in **Fig 1**. Initially, 694 potentially pertinent studies were identified through thorough searches of three databases. Among these, 191 were removed due to duplication. Subsequent screening based on the titles and abstracts resulted in the exclusion of an additional 474 studies that did not align with the aim of the meta-analysis. The full texts of the remaining 29 records underwent independent review by two authors, leading to the removal of a further 19 studies for various reasons, as detailed in **Fig 1**. Ultimately, ten cohort studies remained [14–23] and were considered suitable for subsequent quantitative analyses. A table of all studies identified in the literature search after excluding duplication is shown in **S1 Table,** with reasons for excluding from the meta-analysis if applicable.

### Overview of the studies’ characteristics

All data extracted from the primary research sources are shown in **S2 Table. Table 1** presents the summarized characteristics of the included studies. Overall, five prospective cohort studies [18–20,22,23] and five retrospective cohort studies [14–17,21] were included in the meta-analysis. These studies were published between 2014 and 2023, and were performed in the Netherlands, China, Denmark, Canada, France, Norway, and Mexico. Overall, 833,230 mother-child pair were included. The evaluation of maternal perinatal depression was based on the Brief Symptom Inventory questionnaire [19], Edinburgh Postnatal Depression Scale [18,20], Centre for Epidemiological Studies-Depression [16,21,23], or Global Severity Index [22] in seven studies, and based on database codes[14,15] or self-reported depressive symptoms [17] in three studies. Maternal prenatal depression was evaluated in eight studies [14,17–23], while maternal postpartum depression was evaluated in five studies [15,16,18,20,22]. Accordingly, 40,224 (4.8%) women had prenatal depression. The age for the evaluation of the potential diagnosis of asthma in children varied from two to 10 years. The diagnosis of asthma was based on the clinical evaluation in five studies [16,19–21,23], and was based on self-reporting [15,18,22], prescription records [17], or database codes [14] in the other five studies. Accordingly, 89,645 (10.8%) children were diagnosed with asthma. A univariate regression analysis was performed in one study [20], while multivariate analyses were performed in the other nine studies [14–19,21–23]. Variable maternal, child, and socioeconomic factors were adjusted among the multivariate studies. The NOS of the included studies were six to nine stars, suggesting overall moderate to good study quality (**Table 2**).

**Table 1:** Characteristics of the included studies.

Study	Location	Study design	Number of mother- children pairs included	Methods for validation of depressive symptom in mother	Timing for the evaluating of maternal depressive symptoms	Number of mothers with depressive symptom	Age of children at evaluation (years)	Male offspring (%)	Methods for validation of asthma in children	No. of children with asthma	Variables adjusted
Guxens 2014 [19]	The Netherlands	PC	4848	Clinical evaluation (BSIQ)	Prenatal	388	6	49.1	Clinically diagnosed	291	Maternal age, BMI, smoking during pregnancy, educational level, ethnicity, and parity, parental history of asthma or atopy and pet keeping, children’s sex, preterm birth, birth weight, breast-feeding, day care attendance, secondhand smoke at home, eczema, and lower respiratory tract infections
Wen 2015 [15]	China	RC	19,192	Database codes	Postpartum	3307	5	52.5	Self-reported by caregivers if the child had ever had physician-diagnosed asthma	1267	Maternal age, paternal education, family income, residential area, GA, and children’s birthweight
Liu 2015 [14]	Denmark	RC	733,685	Database codes	Prenatal	21,371	> 3	51.4	Database codes	84,683	Maternal age, maternal parity, maternal social status, maternal smoking during pregnancy, maternal history of asthma, gender of the child, calendar year of birth, and use of other subtypes of antidepressants
Tomfohr 2016 [20]	Canada	PC	1551	Clinical evaluation (EPDS)	Prenatal and postpartum	119	2	51.6	Clinically diagnosed with prescription for asthma	69	None
Zhou 2017 [21]	France	RC	1139	Clinical evaluation (CES-D)	Prenatal	142	5	53.3	Clinically diagnosed	170	Maternal age, study center, maternal education, maternal smoking during pregnancy, maternal BMI, siblings, sex of the children, and family history of allergic diseases
Kozyrskyj 2017 [16]	Canada	RC	1696	Clinical evaluation (CES-D)	Postpartum	331	5~10	49	Clinically diagnosed	237	Children sex, first child, low birthweight, prenatal smoking, maternal asthma, immigrant status, socioeconomic status and family functioning
Magnus 2018 [17]	Norway	RC	63,626	Self-reported major depressive symptoms	Prenatal	13866	7	51.2	Prescription for asthma (codes)	2630	Maternal age, parity, education, prepregnancy BMI, smoking during pregnancy, and maternal history of asthma
R van Meel 2020 [22]	The Netherlands	PC	3640	Clinical evaluation (GSI depressive symptom scale)	Prenatal and postpartum	309	10	48.8	Self-reported and validated by medication records, prescription, and healthcare utilization	213	Maternal age, parity, education level, smoking during pregnancy, BMI at enrolment, history of asthma or atopy and pet keeping, and child’s sex, GA at birth, birth weight, ethnicity, breastfeeding and daycare attendance
Shi 2023 [23]	China	PC	3252	Clinical evaluation (CES-D)	Prenatal	248	2	50.8	Clinically diagnosed	65	Maternal age at delivery, socioeconomic status, maternal parity, exposure to second-hand smoke during pregnancy, family history of asthma, child’s birth weight, and GA
Alcala 2023 [18]	Mexico	PC	601	Clinical evaluation (EPDS)	Prenatal and postpartum	143	4~6	50	Self-reported by caregivers according to the International Study of Asthma and Allergies in Childhood questionnaire	20	Child’s sex, maternal age and education at enrollment, parity, report of a smoker in the home during the second or third trimesters, and average PM2.5 exposure during pregnancy, and average PM2.5 at first year postpartum

PC, prospective cohort; RC, retrospective cohort; BSIQ, Brief Symptom Inventory questionnaire; EPDS, Edinburgh Postnatal Depression Scale; CES-D, Centre for Epidemiological Studies-Depression; BMI, body mass index; GA, gestational age.

**Table 2:** Study quality evaluation using the Newcastle-Ottawa Scale.

Cohort study	Representativeness of the exposed cohort	Selection of the non-exposed cohort	Ascertainment of exposure	Outcome not present at baseline	Control for maternal age	Control for other confounding factors	Assessment of outcome	Sufficient follow-up duration	Adequacy of follow-up of cohorts	Total
Guxens 2014 [19]	1	1	1	1	1	1	1	1	1	9
Wen 2015 [15]	0	1	0	1	1	1	0	1	1	6
Liu 2015 [14]	1	1	0	1	1	1	0	1	1	7
Tomfohr 2016 [20]	1	1	1	1	0	0	1	1	1	7
Zhou 2017 [21]	0	1	1	1	1	1	1	1	1	8
Kozyrskyj 2017 [16]	0	1	1	1	0	1	1	1	1	7
Magnus 2018 [17]	0	1	0	1	1	1	0	1	1	6
R van Meel 2020 [22]	1	1	1	1	1	1	0	1	1	8
Shi 2023 [23]	1	1	1	1	1	1	1	1	1	9
Alcala 2023 [18]	1	1	1	1	1	1	0	1	1	8

### Meta-analysis results

**Fig 2:**
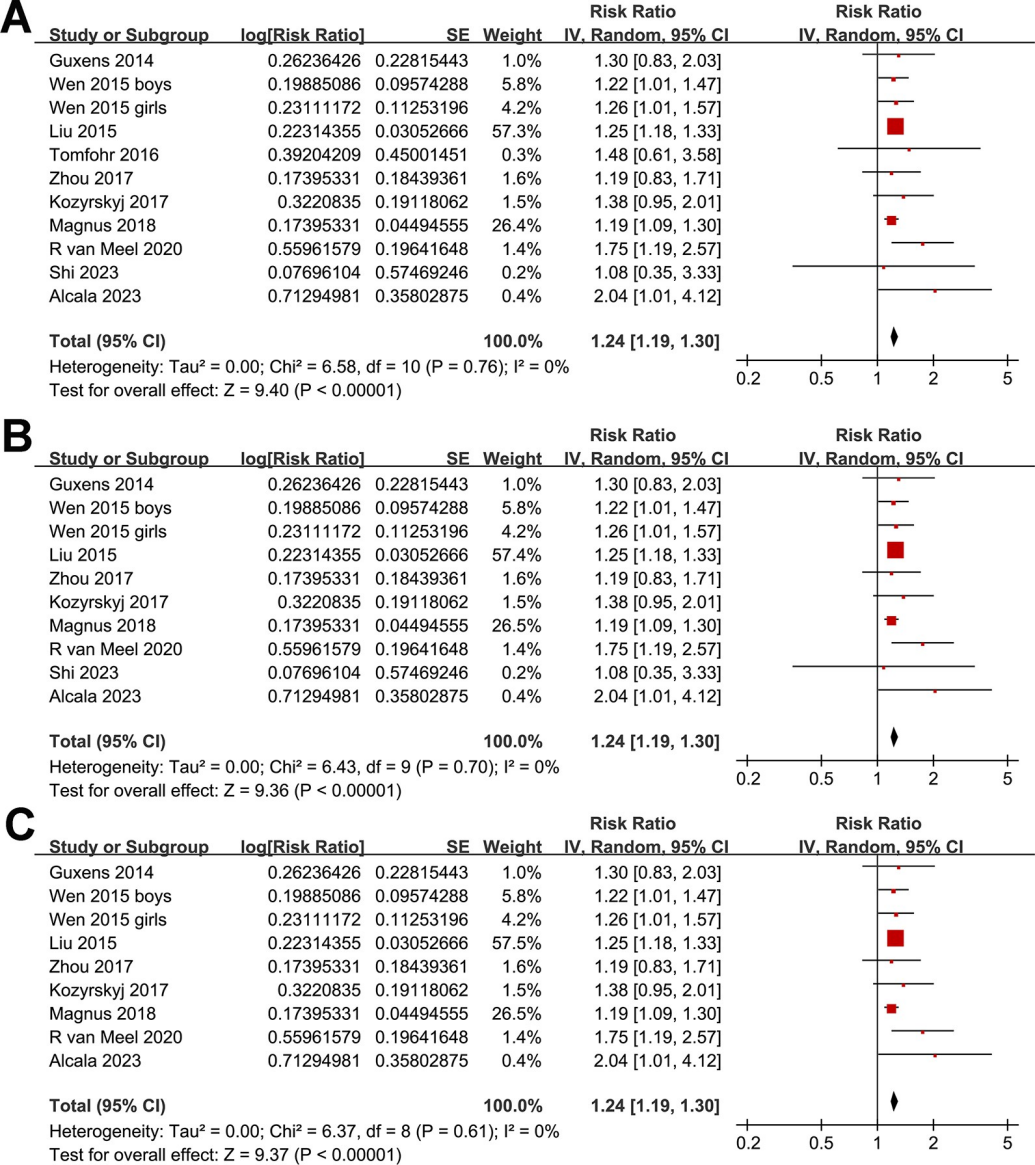
Forest plots for the meta-analysis of the association between maternal perinatal depression and childhood asthma in offspring. A, forest plots for the overall meta-analysis; B, forest plots for the sensitivity analysis limited to studies with multivariate analysis; and C, forest plots for the sensitivity analysis limited to studies with asthma diagnosed in children at least of three years of age.

**Fig 3:**
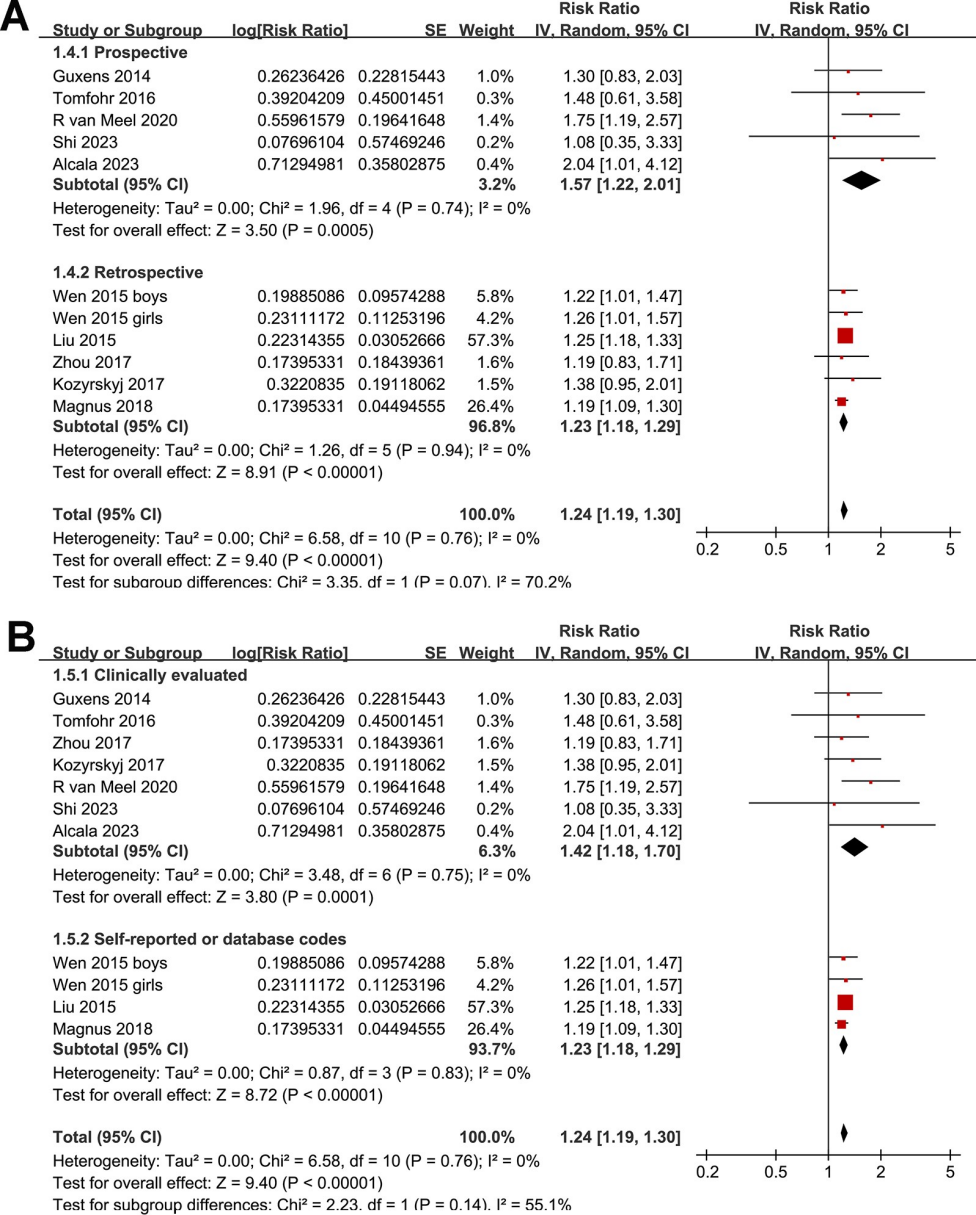
Forest plots for the subgroup analysis of the association between maternal perinatal depression and childhood asthma in offspring. A, forest plots for the subgroup analysis according to study design; and B, forest plots for the subgroup analysis according to methods used to diagnose depression in mothers.

**Fig 4:**
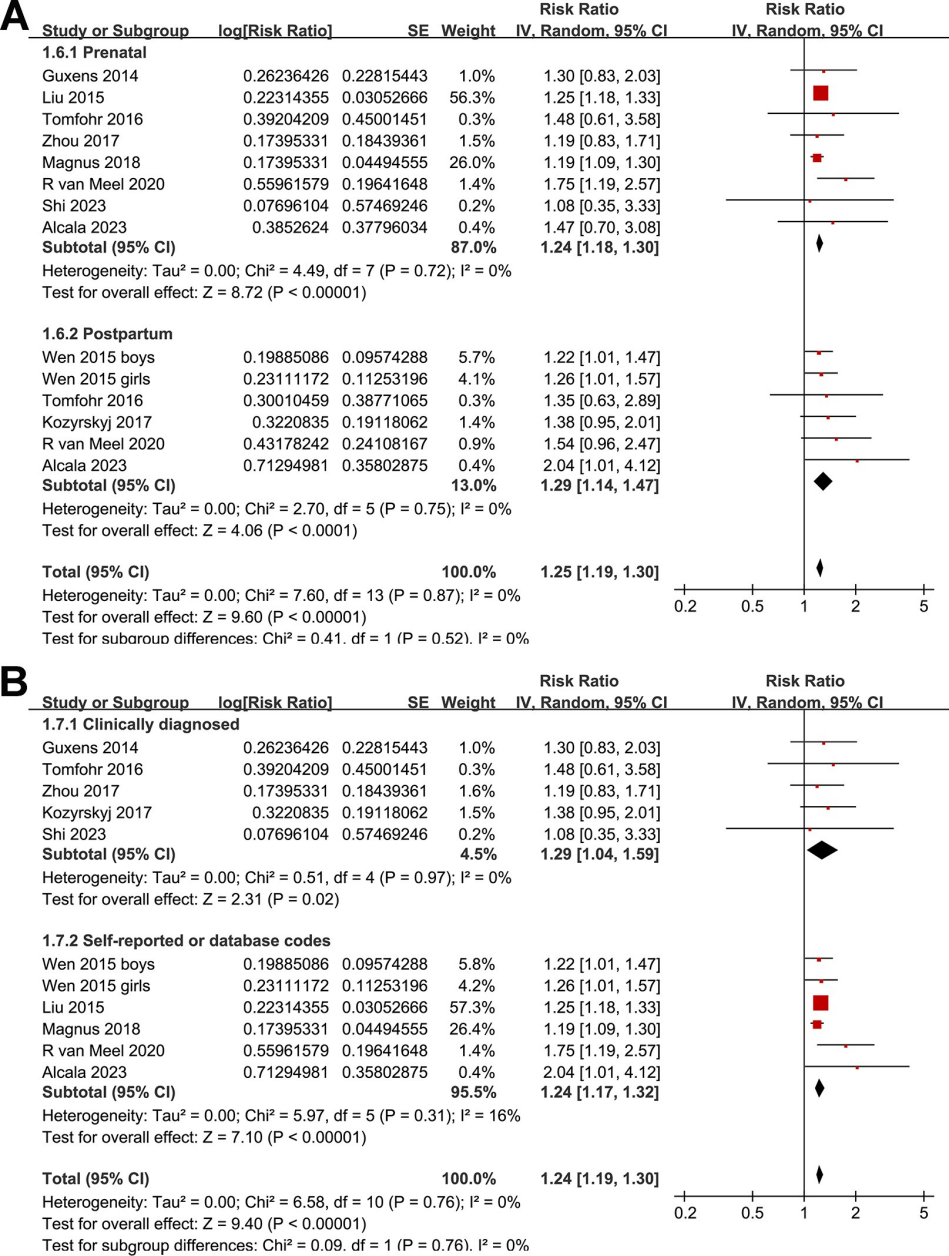
Forest plots for the subgroup analysis of the association between maternal perinatal depression and childhood asthma in offspring. A, forest plots for the subgroup analysis according to timing for evaluating maternal depression; and B, forest plots for the subgroup analysis according to the methods for the diagnosis of asthma in offspring.

**Fig 5:**
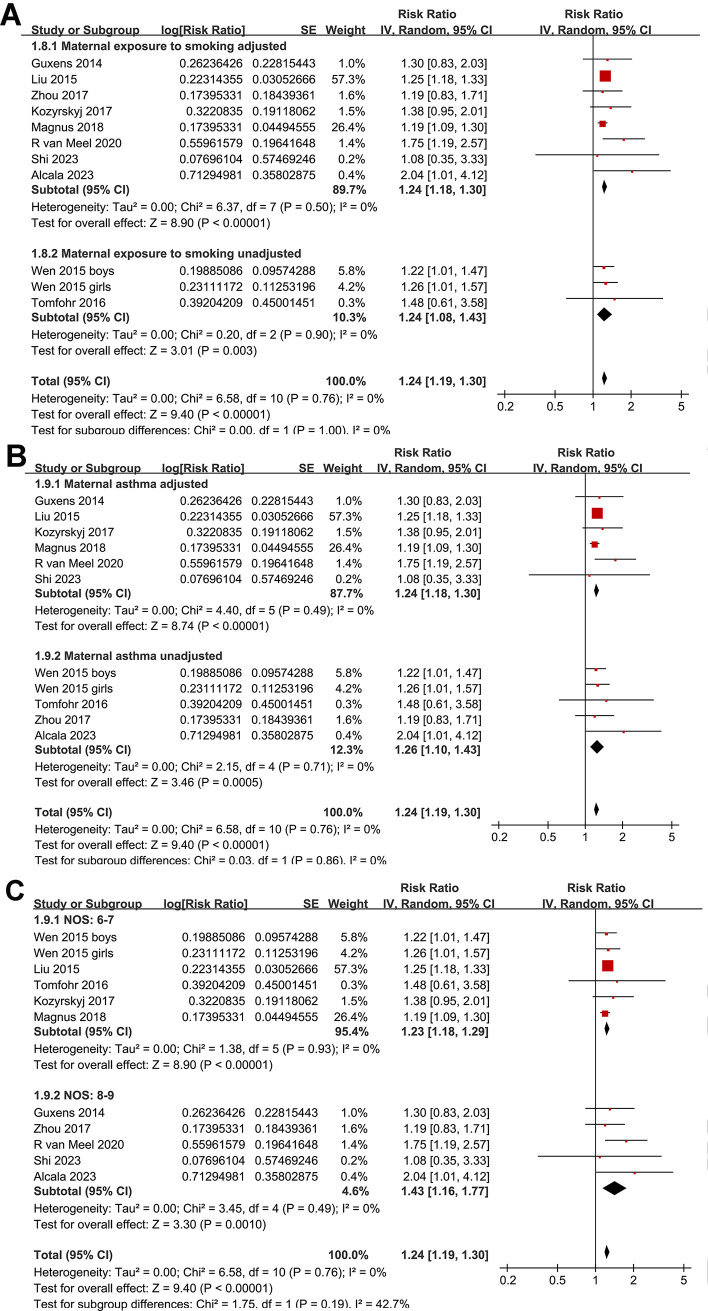
Forest plots for the subgroup analysis of the association between maternal perinatal depression and childhood asthma in offspring. A, forest plots for the adjustment of maternal smoking exposure during pregnancy; B, forest plots for the adjustment of maternal asthma; and C, forest plots for the subgroup analysis according to the study quality scores.

One study presented results based on the gender of the children [15], so these datasets were individually incorporated into the meta-analysis. Pooled results with a random-effects model showed that, compared to women without perinatal depression, perinatal depression in mothers was associated with a significantly increased risk of asthma in offspring (RR: 1.24, 95% CI: 1.19 to 1.30, *p* < 0.001; I^2^ = 0%; **Fig 2A**). Sensitivity analyses conducted by excluding one dataset at a time showed similar results. Further sensitivity analyses restricted to multivariate studies (RR: 1.24, 95% CI: 1.19 to 1.30, *p* < 0.001; I^2^ = 0%; **Fig 2B**) or studies where asthma was diagnosed in children aged three years or older (RR: 1.24, 95% CI: 1.19 to 1.30, *p* < 0.001; I^2^ = 0%; **Fig 2C**) revealed consistent outcomes. Moreover, subgroup analyses showed consistent results in prospective and retrospective studies (**Fig 3A**), in studies with maternal depression evaluated by clinical scales and by self-report or database codes (**Fig 3B**), in studies evaluating prenatal or postpartum depression (**Fig 4A**), in studies with offspring asthma diagnosed by clinical evaluation or self-report or database codes (**Fig 4B**), in studies with or without adjustment of maternal smoking exposure during pregnancy (**Fig 5A**), in studies with or without adjustment for maternal history of asthma (**Fig 5B**), and in studies with different NOS (**Fig 5C**).

### Publication bias evaluation

**Fig 6:**
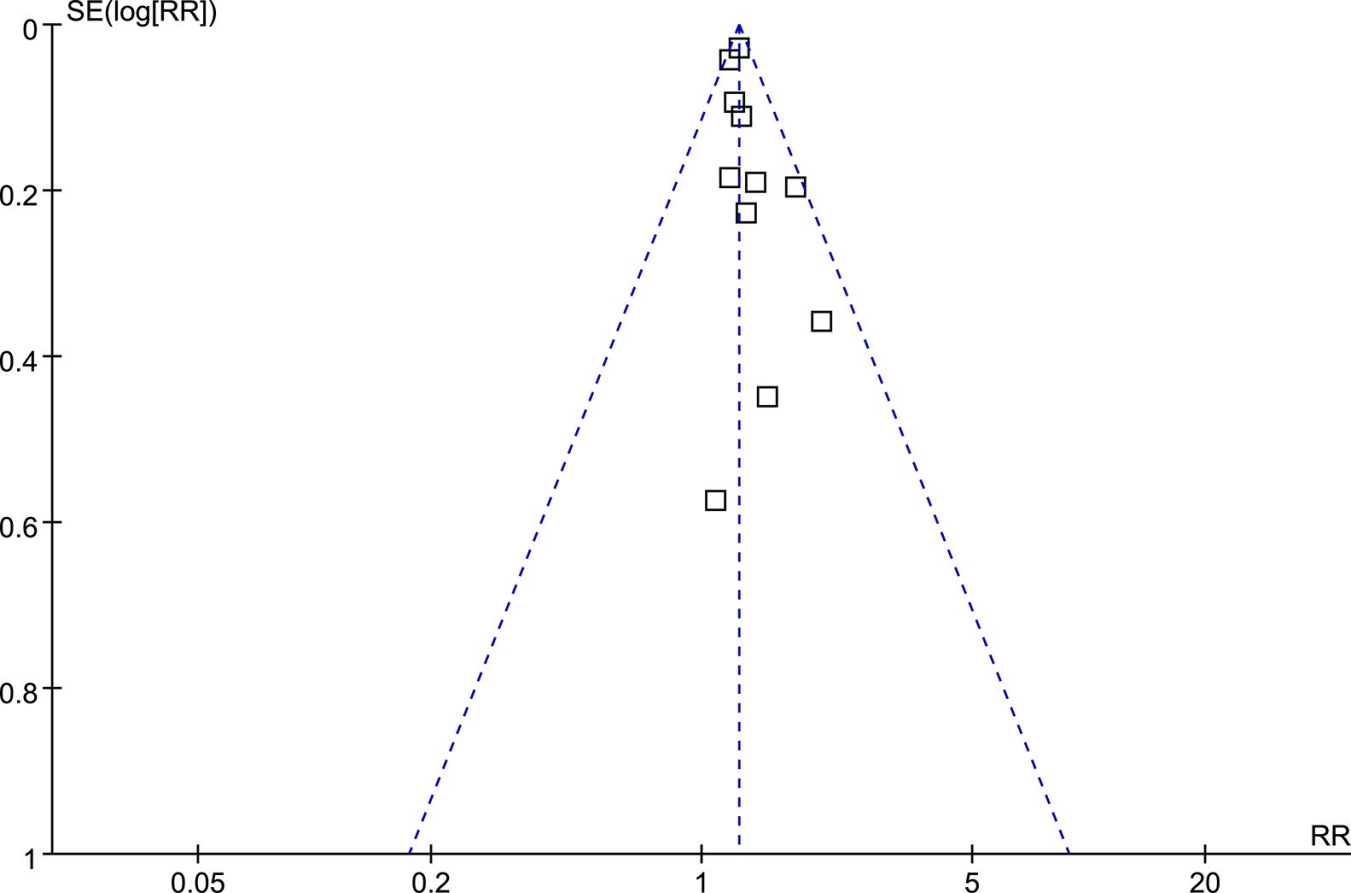
Funnel plots for the publication bias underlying the meta-analysis of the association between maternal perinatal depression and childhood asthma in offspring.

The funnel plots in **Fig 6** display the results of the meta-analysis investigating the link between maternal perinatal depression and offspring asthma risk. The balanced appearance of the funnel plots indicates a minimal chance of publication bias. Additionally, the Egger’s regression test yielded a low risk of publication bias (p = 0.38).

## Discussion

In this comprehensive analysis, we combined the findings of 11 data sets from ten cohort studies and found that women with perinatal depression have a higher likelihood of their offspring developing asthma compared to those without perinatal depression. Additional analyses focusing on multivariate studies and children diagnosed with asthma at age three or older consistently supported these results. Moreover, various subgroup analyses revealed that study characteristics, such as design, methods for assessing maternal depressive symptoms, timing of maternal depression (prenatal or postpartum), offspring asthma diagnosis methods, adjustment for maternal smoking during pregnancy and maternal history of asthma, and differences in study quality scores, did not significantly alter the association between maternal perinatal depression and the risk of asthma in offspring. Overall, our meta-analysis suggests a potential link between maternal perinatal depression and increased risk of asthma in offspring regardless of whether it occurs prenatally or postpartum.

Compared to previous meta-analyses exploring the link between maternal depression and asthma risk in offspring [24,25], our meta-analyses have several advantages. Firstly, we specifically focused on investigating the impact of maternal perinatal depression on the likelihood of asthma in offspring and conducted a comprehensive literature review that identified 10 recent cohort studies relevant to our analysis objectives. Secondly, all the studies included were cohort studies, enabling us to establish a longitudinal association between maternal perinatal depression and the heightened risk of asthma in offspring. Thirdly, sensitivity analysis confined to multivariate studies yielded consistent findings, suggesting that the link between maternal perinatal depression and asthma in offspring may not be influenced by other factors, such as maternal age, gestational age, children’s sex, or socioeconomic circumstances within the family. Finally, multiple predefined subgroup analyses were carried out that further affirmed the strength and reliability of our conclusions.

Our comprehensive analysis has shown a consistent connection between maternal prenatal and postpartum depression and an increased likelihood of asthma in children. However, the specific mechanisms behind these connections are still not fully understood. Previous studies have suggested that maternal prenatal depression might trigger the activation of the hypothalamic-pituitary-adrenal (HPA) axis, leading to excessive cortisol production [34–36]. This excess cortisol is not completely metabolized by the placenta, leading to the release of placental steroids that can cross over to the fetus [34]. These steroids may affect brain development and contribute to airway inflammation and hyper-responsiveness [34,37]. Additionally, higher levels of maternal cortisol can impact fetal immune regulation by shifting the TH1/TH2 lymphocyte balance towards a TH2 response, which could lead to asthma in genetically susceptible children [38]. It is likely that these pathways play a significant role in connecting maternal prenatal depression with asthma onset in offspring. Similarly, the connection between maternal postpartum depression and the risk of asthma in offspring may be linked to dysfunctions in the maternal HPA axis, leading to increased cortisol levels in infants [39,40]. Research has also indicated that infants born to mothers with depressive symptoms show lower concentrations of fecal secretory immunoglobulin A, which can increase their susceptibility to allergic diseases such as asthma [41]. Depressed mothers may struggle with consistent caregiving practices, impacting their child’s exposure to essential stimuli and stressors for immune development, such as a balanced diet, regular medical check-ups, and physical activity [42]. Additionally, maternal depression can negatively influence the home environment, potentially increasing the child’s exposure to environmental allergens or pollutants [43]. This problem could include reduced household cleanliness and management, leading to greater exposure to indoor allergens known to trigger asthma [43]. The quality of maternal-infant interactions is another critical factor, as poor bonding and increased stress exposure in early life, often associated with postnatal maternal depression, can alter immune responses and heighten susceptibility to asthma and other allergic conditions [42,44]. Thus, the relationship between postnatal maternal depression and offspring asthma is complex, involving both direct biological effects and indirect influences through environmental and caregiving factors. Further research is necessary to better understand these pathways and to develop interventions to reduce asthma risk in affected children [44]. However, a previous Swedish population-based study found that cumulative exposure to maternal depression or anxiety, rather than specific critical periods (pre-conception, pregnancy, or postnatal), was most strongly associated with increased risk of childhood asthma [45], suggesting that distinguishing between prenatal and postnatal depression may be less critical in understanding asthma risk.

On the other hand, our subgroup analysis revealed a consistent association between maternal perinatal depression and an elevated risk of asthma in children, even when considering studies that adjusted for maternal exposure to smoking during pregnancy and maternal history of asthma. This suggests that the relationship between maternal depression and asthma in offspring is unlikely to be influenced by smoking, despite the high rate of smoking among people with depression [46] and the established link between maternal smoking in pregnancy and asthma development in children [47]. In addition, although it is well established that asthma and depression could be comorbidities [48], the results of the subgroup analysis suggested that women without a history of asthma who had perinatal depression may also have a higher risk of childhood asthma in their offspring. Finally, reverse causation may exist for the association between perinatal maternal depression and risk of offspring asthma. Early signs of asthma in offspring might influence maternal mental health, and this is particularly relevant in studies where maternal depression is assessed postpartum.

This study has some limitations. Firstly, the mechanisms behind the increased risk of asthma in offspring of mothers with prenatal and postnatal depression may differ considerably. Although a subgroup analysis according to the timing of maternal depression was performed and retrieved similar results, it should be acknowledged that prenatal depression can overstretch the postnatal period, making the analysis of these subgroups more complex. Accordingly, the results of the subgroup analysis should be interpreted with caution. Also, we were unable to perform a subgroup analysis according to the duration of maternal depression during the prenatal period because these data were generally not reported in the included studies. Secondly, five of the studies included were retrospective, which could lead to recall and selection biases in the results. Additionally, in some of the included studies, maternal depression or offspring asthma was confirmed using questionnaires or database codes, potentially impacting result accuracy. Moreover, it is important to acknowledge that depression, anxiety, and stress are often difficult to separate, and similar medications can be given for these conditions, which frequently coexist. This could lead to some misclassification within the included studies, and anxiety-related illnesses might also contribute to the observed outcomes. Furthermore, while sensitivity limited to multivariate analysis consistently indicated an association between maternal perinatal depression and childhood asthma in offspring, there is a chance that residual factors could confound this association. Lastly, as this meta-analysis relied on observational studies only, no causal relationship between maternal perinatal depression and childhood asthma in offspring can be concluded from this meta-analysis.

## Conclusion

In summary, the meta-analysis findings suggest a potential link between maternal perinatal depression and a higher likelihood of childhood asthma in offspring. Further research is necessary to confirm these results and understand the factors underlying this association. These studies underscore the significance of assessing and addressing perinatal depressive symptoms in women, which may be important for reducing the risk of asthma in their offspring.

## Supporting information

S1 Checklist PRISMA 2020 checklist. (DOCX)

S1 Table Studies identified after excluding duplications. (DOC)

S2 Table All data extracted from the primary research sources. (XLSX)
