# Supplementary material for: Perinatal maternal depression and the risk of childhood asthma in offspring: A meta-analysis
Source: PLoS One. 2024 Sep 30;19(9):e0310647. doi: 10.1371/journal.pone.0310647 (PMC11441695; doi:10.1371/journal.pone.0310647)
Supplement: S1 Table — (DOC) [file pone.0310647.s002.doc]

**S1 Table. Excluded studies and the reasons for exclusion**

| **Studies excluded in initial screen with reasons (n = 474)** | |
| --- | --- |
| Reasons | Citations |
| Reviews (n = 12) | 1. Ip S, Chung M, Raman G, Chew P, Magula N, DeVine D, et al. Breastfeeding and maternal and infant health outcomes in developed countries. Evidence report/technology assessment. 2007(153):1-186.  2. Rieder M. If children ruled the pharmaceutical industry: The need for pediatric formulations. Drug News and Perspectives. 2010;23(7):458-64.  3. Annamraju H, Mackillop L. Respiratory disease in pregnancy. Obstetrics, Gynaecology and Reproductive Medicine. 2017;27(4):105-11.  4. Bonham CA, Patterson KC, Strek ME. Asthma Outcomes and Management During Pregnancy. Chest. 2018;153(2):515-27.  5. Rosa MJ, Lee AG, Wright RJ. Evidence establishing a link between prenatal and early-life stress and asthma development. Current Opinion in Allergy and Clinical Immunology. 2018;18(2):148-58.  6. Rusconi F, Gagliardi L. Pregnancy complications and wheezing and asthma in childhood. American Journal of Respiratory and Critical Care Medicine. 2018;197(5):580-8.  7. Wood BL, Brown ES, Lehman HK, Khan DA, Lee MJ, Miller BD. The effects of caregiver depression on childhood asthma: Pathways and mechanisms. Annals of Allergy, Asthma and Immunology. 2018;121(4):421-7.  8. Kaplan A, Szefler SJ, Halpin DMG. Impact of comorbid conditions on asthmatic adults and children. npj Primary Care Respiratory Medicine. 2020;30(1).  9. Anbesaw T, Negash A, Mamaru A, Abebe H, Belete A, Ayano G. Suicidal ideation and associated factors among pregnant women attending antenatal care in Jimma medical center, Ethiopia. PLoS ONE. 2021;16(8 August).  10. Ren Z, Bremer AA, Pawlyk AC. Drug development research in pregnant and lactating women. American Journal of Obstetrics and Gynecology. 2021;225(1):33-42.  11. Teelucksingh S, Govind RM, Dobson R, Ovadia C, Nelson-Piercy C. Treating Vestibular Migraine When Pregnant and Postpartum: Progress, Challenges and Innovations. International Journal of Women's Health. 2023;15((Teelucksingh S.; Govind R.M.; Nelson-Piercy C.) Department of Obstetric Medicine, Guy’s and St. Thomas’ NHS Foundation Trust, London, United Kingdom):321-38.  12. Mudiyanselage SB, Dona SWA, Angeles MR, Majmudar I, Marembo M, Tan EJ, et al. The impact of maternal health on child's health outcomes during the first five years of child's life in countries with health systems similar to Australia: A systematic review. PLoS ONE. 2024;19(3 March). |
| Editorial (n = 4) | 1. Bowman MA, Neale AV. Exciting research studies on practical medical problems and health services delivery. Journal of the American Board of Family Medicine. 2011;24(3):221-3.  2. Isaacs D. Perplexing perinatal practices. Journal of Paediatrics and Child Health. 2018;54(2):113-4.  3. Socolovsky C, Phipatanakul W. Nature and nurture: Lifelong consequences of environmental exposures. Annals of Allergy, Asthma and Immunology. 2019;123(6):532-3.  4. Frost A, Wright RJ. Depression and Asthma: Need to Consider Autonomic Imbalance in Asthma Management. Journal of Allergy and Clinical Immunology: In Practice. 2023;11(2):529-31. |
| Meta-analysis (n = 2) | 1. Flanigan C, Sheikh A, DunnGalvin A, Brew BK, Almqvist C, Nwaru BI. Prenatal maternal psychosocial stress and offspring's asthma and allergic disease: A systematic review and meta-analysis. Clinical and Experimental Allergy. 2018;48(4):403-14.  2. Pierce M, Hope HF, Kolade A, Gellatly J, Osam CS, Perchard R, et al. Effects of parental mental illness on children's physical health: Systematic review and meta-analysis. British Journal of Psychiatry. 2020;217(1):354-63. |
| Irrelevant studies (n = 456) | 1. Breen TW, Janzen JA. Pulmonary hypertension and cardiomyopathy: Anaesthetic management for Caesarean section. Canadian Journal of Anaesthesia. 1991;38(7):895-9.  2. Lau C, Cameron AM, Antolick LL, Stanton ME. Repeated maternal separation in the neonatal rat: Cellular mechanisms contributing to brain growth sparing. Journal of Developmental Physiology. 1992;17(6):265-76.  3. Weitzman M, Gortmaker S, Sobol A. Maternal smoking and behavior problems of children. Pediatrics. 1992;90(3 I):342-9.  4. Kaaja R, Julkunen H, Ammala P, Palosuo T, Kurki P. Intravenous immunogobulin treatment of pregnant patients with recurrent pregnancy losses associated with antiphospholipid antibodies. Acta Obstetricia et Gynecologica Scandinavica. 1993;72(1):63-6.  5. Eisenfeld L, Rosenkrantz TS, Block C, Burke G, Phillips F, Herson V, et al. Effect of corticosteroids on the maturation of neutrophil motility in very low birthweight neonates. American Journal of Perinatology. 1994;11(2):163-6.  6. Emery MJ, Hlastala MP, Matsumoto AM. Depression of hypercapnic ventilatory drive by testosterone in the sleeping infant primate. Journal of Applied Physiology. 1994;76(4):1786-93.  7. Baker D, North K. Does employment improve the health of lone mothers? Social Science and Medicine. 1999;49(1):121-31.  8. Piazze JJ, Anceschi MM, Maranghi L, Brancato V, Marchiani E, Cosmi EV. Fetal lung maturity in pregnancies complicated by insulin-dependent and gestational diabetes: A matched cohort study. European Journal of Obstetrics and Gynecology and Reproductive Biology. 1999;83(2):145-50.  9. Singer LT, Salvator A, Guo S, Collin M, Lilien L, Baley J. Maternal psychological distress and parenting stress after the birth of a very low-birth-weight infant. JAMA. 1999;281(9):799-805.  10. Mukherjee JS, Shin S, Furin J, Rich ML, Léandre F, Joseph JK, et al. New challenges in the clinical management of drug-resistant tuberculosis. Infectious Diseases in Clinical Practice. 2002;11(6):329-39.  11. Sadick NS. A practitioner's 10-year experience with isotretinoin and side effect profiles. International Journal of Cosmetic Surgery and Aesthetic Dermatology. 2002;4(2):89-94.  12. Conwell LS, O'Callaghan MJ, Andersen MJ, Bor W, Najman JM, Williams GM. Early adolescent smoking and a web of personal and social disadvantage. Journal of Paediatrics and Child Health. 2003;39(8):580-5.  13. Craig-McFeely PM, Wilton LV, Soriano JB, Maier WC, Shakir SAW. Prospective observational cohort safety study to monitor the introduction of a non-CFC formulation of salbutamol with HFA134a in England. International Journal of Clinical Pharmacology and Therapeutics. 2003;41(2):67-76.  14. Felt-Lisk S, Gold MR. Do quality improvement strategies for Medicaid enrollees differ in Medicaid-dominant versus commercial managed care organizations? American Journal of Managed Care. 2003;9(12):806-16.  15. Fox HB, McManus MA, Reichman MB. Private health insurance for adolescents: Is it adequate? Journal of Adolescent Health. 2003;32(6 SUPPL.):12-24.  16. Timonen M, Jokelainen J, Herva A, Zitting P, Meyer-Rochow VB, Räsänen P. Presence of atopy in first-degree relatives as a predictor of a female proband's depression: Results from the Northern Finland 1966 Birth Cohort. Journal of Allergy and Clinical Immunology. 2003;111(6):1249-54.  17. Al-Asmary SM, Abdel-Fattah MM, Asal ARA, Al-Helali NS, Al-Jabban TM, Arafa MA. Emotional and behavioral problems among male Saudi schoolchildren and adolescents. Neurosciences. 2004;9(4):299-306.  18. Kang W, Tan KH. Implant contraception in Singaporean women: One decade of experience in KK Women's and Children's Hospital. Singapore Medical Journal. 2004;45(10):482-6.  19. Leah Klam S, Leduc L. Management Options for Preterm Labour in Canada. Journal of Obstetrics and Gynaecology Canada. 2004;26(4):339-45.  20. Lutfi S, Allen VM, Fahey J, O'Connell CM, Vincer MJ. Twin-twin transfusion syndrome: A population-based study. Obstetrics and Gynecology. 2004;104(6):1289-97.  21. Pace WD, Dickinson LM, Staton EW. Seasonal variation in diagnoses and visits to family physicians. Annals of Family Medicine. 2004;2(5):411-7.  22. Babu KM, McCormick MA, Bird SB. Pediatric dietary supplement use - An update. Clinical Pediatric Emergency Medicine. 2005;6(2):85-92.  23. Chambers CD, Hernandez-Diaz S, Van Marter LJ, Werler MM, Louik C, Jones KL, et al. Selective serotonin-reuptake inhibitors and risk of persistent pulmonary hypertension of the newborn. New England Journal of Medicine. 2006;354(6):579-87.  24. Gonsalves L, Schuermeyer I. Treating depression in pregnancy: Practical suggestions. Cleveland Clinic Journal of Medicine. 2006;73(12):1098-104.  25. Mahadevan U, Kane S. American Gastroenterological Association Institute Technical Review on the Use of Gastrointestinal Medications in Pregnancy. Gastroenterology. 2006;131(1):283-311.  26. Miyake Y, Sasaki S, Tanaka K, Yokoyama T, Ohya Y, Fukushima W, et al. Dietary folate and vitamins B12, B6, and B2 intake and the risk of postpartum depression in Japan: The Osaka Maternal and Child Health Study. Journal of Affective Disorders. 2006;96(1-2):133-8.  27. Miyake Y, Sasaki S, Yokoyama T, Tanaka K, Ohya Y, Fukushima W, et al. Risk of postpartum depression in relation to dietary fish and fat intake in Japan: The Osaka Maternal and Child Health Study. Psychological Medicine. 2006;36(12):1727-35.  28. Shaw M, Lawlor DA, Najman JM. Teenage children of teenage mothers: Psychological, behavioural and health outcomes from an Australian prospective longitudinal study. Social Science and Medicine. 2006;62(10):2526-39.  29. A look back at pharmaceuticals in 2006: Aggressive advertising cannot hide the absence of therapeutic advances. Prescrire International. 2007;16(88):80-6.  30. Breggin PR, Breggin G. Exposure to SSRI antidepressants in utero causes birth defects, neonatal withdrawal symptoms and brain damage. International Journal of Risk and Safety in Medicine. 2007;19(4):203-7.  31. Lennestål R, Källén B. Delivery outcome in relation to maternal use of some recently introduced antidepressants. Journal of Clinical Psychopharmacology. 2007;27(6):607-13.  32. Tata LJ, Lewis SA, McKeever TM, Smith CJP, Doyle P, Smeeth L, et al. A comprehensive analysis of adverse obstetric and pediatric complications in women with asthma. American Journal of Respiratory and Critical Care Medicine. 2007;175(10):991-7.  33. Vedam S, Goff M, Marnin VN. Closing the Theory-Practice Gap: Intrapartum Midwifery Management of Planned Homebirths. Journal of Midwifery and Women's Health. 2007;52(3):291-300.  34. SSRI antidepressants and persistent pulmonary hypertension in newborns. Prescrire International. 2008;17(96):156.  35. Bardeguez AD, Lindsey JC, Shannon M, Tuomala RE, Cohn SE, Smith E, et al. Adherence to antiretrovirals among US women during and after pregnancy. Journal of Acquired Immune Deficiency Syndromes. 2008;48(4):408-17.  36. Belik J. Fetal and Neonatal Effects of Maternal Drug Treatment for Depression. Seminars in Perinatology. 2008;32(5):350-4.  37. Breggin PR, Breggin G. Exposure to SSRI antidepressants in utero causes birth defects, neonatal withdrawal symptoms, and brain damage. Ethical Human Psychology and Psychiatry. 2008;10(1):5-9.  38. Chatterjee S, Kotelchuck M, Sambamoorthi U. Prevalence of Chronic Illness in Pregnancy, Access to Care, and Health Care Costs. Implications for Interconception Care. Women's Health Issues. 2008;18(6 SUPPL.):S107-S16.  39. Gupta N, Corrado S, Goldstein M. Hormonal contraception for the adolescent. Pediatrics in Review. 2008;29(11):386-97.  40. Hoskins KE, Tita ATN, Biggio JR, Ramsey PS. Pregnancy and active Huntington disease: A rare combination. Journal of Perinatology. 2008;28(2):156-7.  41. Leslie RC, Shepherd MD, Simmons SC. Use of a diagnosis-based risk adjustment model to estimate costs of indigent care in a community at medicaid reimbursement rates. Journal of Medical Economics. 2008;11(4):585-600.  42. Murakami K, Miyake Y, Sasaki S, Tanaka K, Yokoyama T, Ohya Y, et al. Dietary glycemic index and load and the risk of postpartum depression in Japan: The Osaka Maternal and Child Health Study. Journal of Affective Disorders. 2008;110(1-2):174-9.  43. Tremlett HL, Oger J. Ten years of adverse drug reaction reports for the multiple sclerosis immunomodulatory therapies: A Canadian perspective. Multiple Sclerosis. 2008;14(1):94-105.  44. Einarson A, Choi J, Einarson TR, Koren G. Incidence of major malformations in infants following antidepressant exposure in pregnancy: Results of a large prospective cohort study. Canadian Journal of Psychiatry. 2009;54(4):242-6.  45. Ferguson S, Allen VM, Craig C, Allen AC, Dodds L. Timing of indicated delivery after antenatal steroids in preterm pregnancies with severe hypertension. Hypertension in Pregnancy. 2009;28(1):63-75.  46. Koren G, Boucher N. Adverse effects in neonates exposed to SSRIs and SNRI in late gestation. Canadian Journal of Clinical Pharmacology. 2009;16(1):e66-e7.  47. Lung FW, Chiang TL, Lin SJ, Shu BC. Parental mental health and child development from six to thirty-six months in a birth cohort study in Taiwan. Journal of Perinatal Medicine. 2009;37(4):397-402.  48. Makrides M. Is there a dietary requirement for DHA in pregnancy? Prostaglandins Leukotrienes and Essential Fatty Acids. 2009;81(2-3):171-4.  49. Mesquita AR, Wegerich Y, Patchev AV, Oliveira M, Leão P, Sousa N, et al. Glucocorticoids and neuro- and behavioural development. Seminars in Fetal and Neonatal Medicine. 2009;14(3):130-5.  50. Yonkers KA, Wisner KL, Stewart DE, Oberlander TF, Dell DL, Stotland N, et al. The management of depression during pregnancy: a report from the American Psychiatric Association and the American College of Obstetricians and Gynecologists. General Hospital Psychiatry. 2009;31(5):403-13.  51. Aziz S, Qamar R, Ahmed I, Imran K, Masroor M, Rajper J, et al. Treatment profile of hepatitis C patients - A comparison of interferon alpha 2a and 2b treatment regimes. Journal of the College of Physicians and Surgeons Pakistan. 2010;20(9):581-5.  52. Dreger LC, Kozyrskyj AL, HayGlass KT, Becker AB, MacNeil BJ. Lower cortisol levels in children with asthma exposed to recurrent maternal distress from birth. Journal of Allergy and Clinical Immunology. 2010;125(1-3):116-22.  53. Fishell A. Depression and anxiety in pregnancy. Canadian Journal of Clinical Pharmacology. 2010;17(3):e363-e9.  54. Hayatbakhsh MR, Najman JM, Clavarino A, Bor W, Williams GM, O'Callaghan MJ. Association of psychiatric disorders, asthma and lung function in early adulthood. Journal of Asthma. 2010;47(7):786-91.  55. King NMA, Chambers J, O'Donnell K, Jayaweera SR, Williamson C, Glover VA. Anxiety, depression and saliva cortisol in women with a medical disorder during pregnancy. Archives of Women's Mental Health. 2010;13(4):339-45.  56. Nezvalová-Henriksen K, Spigset O, Nordeng H. Triptan exposure during pregnancy and the risk of major congenital malformations and adverse pregnancy outcomes: Results from the Norwegian mother and child cohort study. Headache. 2010;50(4):563-75.  57. Rabaiotti E, Sigismondi C, Montoli S, Mangili G, Candiani M, Viganò R. Management of locally advanced cervical cancer in pregnancy: A case report. Tumori. 2010;96(4):623-6.  58. Sani MN, Malekiyan A, Jamnani AN. Coincidence of cystic fibrosis in mother and her child related to infertility. International Journal of Fertility and Sterility. 2010;4(2):85-7.  59. Singer LT, Fulton S, Kirchner HL, Eisengart S, Lewis B, Short E, et al. Longitudinal predictors of maternal stress and coping after very low-birth-weight birth. Archives of Pediatrics and Adolescent Medicine. 2010;164(6):518-24.  60. Thornton D, Guendelman S, Hosang N. Obstetric complications in women with diagnosed mental illness: The relative success of California's county mental health system. Health Services Research. 2010;45(1):246-64.  61. Veeram Reddy SR, Singh HR. Chest pain in children and adolescents. Pediatrics in Review. 2010;31(1):e1-e9.  62. Yolton K, Xu Y, Khoury J, Succop P, Lanphear B, Beebe DW, et al. Associations between secondhand smoke exposure and sleep patterns in children. Pediatrics. 2010;125(2):e261-e8.  63. Skin-lightening cosmetics: Frequent, potentially severe adverse effects. Prescrire International. 2011;20(119):209-15.  64. Bandiera FC, Kalaydjian Richardson A, Lee DJ, He JP, Merikangas KR. Secondhand smoke exposure and mental health among children and adolescents. Archives of Pediatrics and Adolescent Medicine. 2011;165(4):332-8.  65. Buist AE, Bilszta J. Perinatal mental illness: Identifying and managing women at risk. Medicine Today. 2011;12(1):64-8.  66. Fenger-Grøn J, Thomsen M, Andersen KS, Nielsen RG. Paediatric outcomes following intrauterine exposure to serotonin reuptake inhibitors - A systematic review. Danish Medical Bulletin. 2011;58(9).  67. Ghidini A, Simonson MR. Pregnancy after spinal cord injury: A review of the literature. Topics in Spinal Cord Injury Rehabilitation. 2011;16(3):93-103.  68. Haas DM, Gallauresi B, Shields K, Zeitlin D, Clark SM, Hebert MF, et al. Pharmacotherapy and pregnancy: Highlights from the third international conference for individualized pharmacotherapy in pregnancy. Clinical and Translational Science. 2011;4(3):204-9.  69. Hofer AN, Abraham JM, Moscovice I. Expansion of coverage under the patient protection and affordable care act and primary care utilization. Milbank Quarterly. 2011;89(1):69-89.  70. Kamiński K, Wietrak E, Popiel M. The role of docosahexaenoic acid (DHA) in pregnancy. How much should we use? Ginekologia i Poloznictwo. 2011;21(3):113-27.  71. Kondo N, Suda Y, Nakao A, Oh-Oka K, Suzuki K, Ishimaru K, et al. Maternal psychosocial factors determining the concentrations of transforming growth factor-beta in breast milk. Pediatric Allergy and Immunology. 2011;22(8):853-61.  72. Murphy KE, Hannah ME, Willan AR, Ohlsson A, Kelly EN, Matthews SG, et al. Maternal Side-Effects After Multiple Courses of Antenatal Corticosteroids (MACS): The Three- Month Follow-Up of Women in the Randomized Controlled Trial of MACS for Preterm Birth Study. Journal of Obstetrics and Gynaecology Canada. 2011;33(9):909-21.  73. Nakhai-Pour HR, Broy P, Sheehy O, Beŕard A. Use of nonaspirin nonsteroidal anti-inflammatory drugs during pregnancy and the risk of spontaneous abortion. CMAJ Canadian Medical Association Journal. 2011;183(15):1713-20.  74. Özkan H, Çetinkaya M, Köksal N, Yapici Ş. Severe fetal valproate syndrome: Combination of complex cardiac defect, multicystic dysplastic kidney, and trigonocephaly. Journal of Maternal-Fetal and Neonatal Medicine. 2011;24(3):521-4.  75. Suglia SF, Chambers EC, Rosario A, Duarte CS. Asthma and obesity in three-year-old urban children: Role of sex and home environment. Journal of Pediatrics. 2011;159(1):14-20.e1.  76. Urato AC. Antidepressants and pregnancy: Continued evidence of harm-still no evidence of benefit. Ethical Human Psychology and Psychiatry. 2011;13(3):190-3.  77. Yonkers KA, Vigod S, Ross LE. Diagnosis, pathophysiology, and management of mood disorders in pregnant and postpartum women. Obstetrics and Gynecology. 2011;117(4):961-77.  78. Arch JJ, Dimidjian S, Chessick C. Are exposure-based cognitive behavioral therapies safe during pregnancy? Archives of Women's Mental Health. 2012;15(6):445-57.  79. Di Renzo GC, Giardina I, Clerici G, Mattei A, Alajmi AH, Gerli S. The role of progesterone in maternal and fetal medicine. Gynecological Endocrinology. 2012;28(11):925-32.  80. Drake AL, Roxby AC, Kiarie J, Richardson BA, Wald A, John-Stewart G, et al. Infant safety during and after maternal valacyclovir therapy in conjunction with antiretroviral HIV-1 prophylaxis in a randomized clinical trial. PLoS ONE. 2012;7(4).  81. Erni K, Shaqiri-Emini L, La Marca R, Zimmermann R, Ehlert U. Psychobiological effects of prenatal glucocorticoid exposure in 10-year-old-children. Frontiers in Psychiatry. 2012;3(DEC).  82. García-Huidobro D, Puschel K, Soto G. Family functioning style and health: Opportunities for health prevention in primary care. British Journal of General Practice. 2012;62(596):e198-e203.  83. Krüger S. Psychopharmacological treatment of mood and anxiety disorders during pregnancy. 2012. p. 279-305.  84. Lauer B, Spector N. Vitamins. Pediatrics in Review. 2012;33(8):339-52.  85. Laurence V, Rousset-Jablonski C. Contraception and cancer treatment in young persons. 2012. p. 41-60.  86. Madden T, Eisenberg DL, Zhao Q, Buckel C, Secura GM, Peipert JF. Continuation of the etonogestrel implant in women undergoing immediate postabortion placement. Obstetrics and Gynecology. 2012;120(5):1053-9.  87. Naz T, Hassan L, Rafique I. Magnesium sulphate therapy in eclampsia: A 5 years experience at a teaching hospital. JPMI - Journal of Postgraduate Medical Institute. 2012;26(1):84-90.  88. Sánchez-Borges M, Asero R, Ansotegui IJ, Baiardini I, Bernstein JA, Canonica GW, et al. Diagnosis and treatment of urticaria and angioedema: A worldwide perspective. World Allergy Organization Journal. 2012;5(11):125-47.  89. Sie SD, Wennink JMB, Van Driel JJ, Te Winkel AGW, Boer K, Casteelen G, et al. Maternal use of SSRIs, SNRIs and NaSSAs: Practical recommendations during pregnancy and lactation. Archives of Disease in Childhood: Fetal and Neonatal Edition. 2012;97(6):F472-F6.  90. Singh RR, Cuffe JS, Moritz KM. Short- and long-term effects of exposure to natural and synthetic glucocorticoids during development. Clinical and Experimental Pharmacology and Physiology. 2012;39(11):979-89.  91. Šumilo D, Kurinczuk JJ, Redshaw ME, Gray R. Prevalence and impact of disability in women who had recently given birth in the UK. BMC Pregnancy and Childbirth. 2012;12((Šumilo D., dana.sumilo@npeu.ox.ac.uk; Kurinczuk J.J., jenny.kurinczuk@npeu.ox.ac.uk; Redshaw M.E., maggie.redshaw@npeu.ox.ac.uk; Gray R., ron.gray@npeu.ox.ac.uk) Policy Research Unit in Maternal Health and Care, National Perinatal Epidemiology Unit, Department of Public Health, University of Oxford, Old Road Campus, Oxford, OX3 7LF, United Kingdom).  92. Sung V, Hiscock H, Tang M, Mensah FK, Heine RG, Stock A, et al. Probiotics to improve outcomes of colic in the community: Protocol for the Baby Biotics randomised controlled trial. BMC Pediatrics. 2012;12((Sung V., valerie.sung@rch.org.au; Hiscock H., harriet.hiscock@rch.org.au; York E., elissa.york@mcri.edu.au; Wake M., melissa.wake@rch.org.au) Centre for Community Child Health, Royal Children's Hospital, Parkville, Australia).  93. Toohey J. Depression during pregnancy and postpartum. Clinical Obstetrics and Gynecology. 2012;55(3):788-97.  94. Vos T, Flaxman AD, Naghavi M, Lozano R, Michaud C, Ezzati M, et al. Years lived with disability (YLDs) for 1160 sequelae of 289 diseases and injuries 1990-2010: A systematic analysis for the Global Burden of Disease Study 2010. The Lancet. 2012;380(9859):2163-96.  95. Bain ES, Middleton PF, Crowther CA. Maternal adverse effects of different antenatal magnesium sulphate regimens for improving maternal and infant outcomes: A systematic review. BMC Pregnancy and Childbirth. 2013;13((Bain E.S., emily.bain@adelaide.edu.au; Middleton P.F., philippa.middleton@adelaide.edu.au; Crowther C.A., caroline.crowther@adelaide.edu.au) Australian Research Centre for Health of Women and Babies, Robinson Institute, Discipline of Obstetrics and Gynaecology, School of Paediatrics and Reproductive Health, The University of Adelaide, 72 King William Road, Adelaide, SA, Australia).  96. Hines M, Lyseng-Williamson KA, Deeks ED. 17 α-hydroxyprogesterone caproate (Makena®): A guide to its use in the prevention of preterm birth. Clinical Drug Investigation. 2013;33(3):223-7.  97. Howell LJ. The Garbose Family Special Delivery Unit: A new paradigm for maternal-fetal and neonatal care. Seminars in Pediatric Surgery. 2013;22(1):3-9.  98. Korkeila J, Salokangas RKR, Heinimaaa M, Svirskis T, Laine T, Ruhrmann S, et al. Physical illnesses, developmental risk factors and psychiatric diagnoses among subjects at risk of psychosis. European Psychiatry. 2013;28(3):135-40.  99. Liu S, Joseph KS, Lisonkova S, Rouleau J, Van Den Hof M, Sauve R, et al. Association between maternal chronic conditions and congenital heart defects: A population-based cohort study. Circulation. 2013;128(6):583-9.  100. Lowenthal A, Lal A, Tierney ESS, Tacy TA. Tricuspid atresia with progressive ductal restriction in a fetus. Pediatric Cardiology. 2013;34(6):1499-501.  101. Mathisen SE, Glavin K, Lien L, Lagerløv P. Prevalence and risk factors for postpartum depressive symptoms in Argentina: A cross-sectional study. International Journal of Women's Health. 2013;5(1):787-93.  102. O'Byrne PM, Pedersen S, Schatz M, Thoren A, Ekholm E, Carlsson LG, et al. The poorly explored impact of uncontrolled asthma. Chest. 2013;143(2):511-23.  103. Radosevich MA, Finegold H, Goldfarb W, Troianos C. Anesthetic management of the pregnant burn patient: Excision and grafting to emergency Cesarean section. Journal of Clinical Anesthesia. 2013;25(7):582-6.  104. Rodríguez-Martínez CE, Sossa-Briceño MP, Castro-Rodriguez JA. Predictors of hospitalization for asthma in children: Results of a 1-year prospective study. Pediatric Pulmonology. 2013((Rodríguez-Martínez C.E., carerodriguezmar@unal.edu.co) Department of Pediatrics, School of Medicine Universidad Nacional de Colombia Bogota Colombia).  105. Shah PE, Robbins N, Coelho RB, Poehlmann J. The paradox of prematurity: The behavioral vulnerability of late preterm infants and the cognitive susceptibility of very preterm infants at 36 months post-term. Infant Behavior and Development. 2013;36(1):50-62.  106. Stephansson O, Kieler H, Haglund B, Artama M, Engeland A, Furu K, et al. Selective serotonin reuptake inhibitors during pregnancy and risk of stillbirth and infant mortality. JAMA. 2013;309(1):48-54.  107. Šumilo D, Kurinczuk JJ, Redshaw ME, Gray R. Association between limiting longstanding illness in mothers and their children: Findings from the UK Millennium Cohort Study. BMJ Open. 2013;3(12).  108. Tordjman S, Anderson GM, Cohen D, Kermarrec S, Carlier M, Touitou Y, et al. Presence of autism, hyperserotonemia, and severe expressive language impairment in Williams-Beuren syndrome. Molecular Autism. 2013;4(1).  109. van Zyl-Smit RN, Allwood B, Stickells D, Symons G, Abdool-Gaffar S, Murphy K, et al. South African tobacco smoking cessation clinical practice guideline. South African Medical Journal. 2013;103(11):869-76.  110. Werder E, Mendola P, Männistö T, O'Loughlin J, Laughon SK. Effect of maternal chronic disease on obstetric complications in twin pregnancies in a United States cohort. Fertility and Sterility. 2013;100(1):142-9.e2.  111. Ansary A, Ibhanesebhor S, Manjunatha C. Myoclonic seizures in a preterm baby: Is this a presentation of venlafaxine withdrawal? Singapore Medical Journal. 2014;55(4):e57-e9.  112. Ban L, Gibson JE, West J, Fiaschi L, Sokal R, Smeeth L, et al. Maternal depression, antidepressant prescriptions, and congenital anomaly risk in offspring: A population-based cohort study. BJOG: An International Journal of Obstetrics and Gynaecology. 2014;121(12):1471-81.  113. Bourke CH, Stowe ZN, Owens MJ. Prenatal antidepressant exposure: Clinical and preclinical findings. Pharmacological Reviews. 2014;66(2):435-65.  114. Chandane PG, Shah I. Fetal valproate syndrome. Indian Journal of Human Genetics. 2014;20(2):187-8.  115. Chang HY, Keyes KM, Lee KS, Choi IA, Kim SJ, Kim KW, et al. Prenatal maternal depression is associated with low birth weight through shorter gestational age in term infants in Korea. Early Human Development. 2014;90(1):15-20.  116. Chrousos G. Video Q & A: The impact of stress. An interview with George Chrousos. BMC Medicine. 2014;12(1).  117. Gaillard A, Le Strat Y, Mandelbrot L, Keïta H, Dubertret C. Predictors of postpartum depression: Prospective study of 264 women followed during pregnancy and postpartum. Psychiatry Research. 2014;215(2):341-6.  118. Gonçalves H, Assunção MCF, Wehrmeister FC, Oliveira IO, Barros FC, Victora CG, et al. Cohort profile update: The 1993 Pelotas (Brazil) birth cohort follow-up visits in adolescence. International Journal of Epidemiology. 2014;43(4):1082-8.  119. Hanley GE, Oberlander TF. The effect of perinatal exposures on the infant: Antidepressants and depression. Best Practice and Research: Clinical Obstetrics and Gynaecology. 2014;28(1):37-48.  120. Hinkle SN, Albert PS, Mendola P, Sjaarda LA, Boghossian NS, Yeung E, et al. Differences in risk factors for incident and recurrent small-for- gestational-age birthweight: A hospital-based cohort study. BJOG: An International Journal of Obstetrics and Gynaecology. 2014;121(9):1080-9.  121. Jonklaas J, Bianco AC, Bauer AJ, Burman KD, Cappola AR, Celi FS, et al. Guidelines for the treatment of hypothyroidism: Prepared by the American thyroid association task force on thyroid hormone replacement. Thyroid. 2014;24(12):1670-751.  122. Jordan B, Franich-Ray C, Albert N, Anderson V, Northam E, Cochrane A, et al. Early mother-infant relationships after cardiac surgery in infancy. Archives of Disease in Childhood. 2014;99(7):641-5.  123. Judge C, O'Donovan C, Callaghan G, Gaoatswe G, O'Shea D. Gender dysphoria - prevalence and co-morbidities in an Irish adult population. Frontiers in Endocrinology. 2014;5(JUN).  124. Ko MC, Hung YH, Ho PY, Yang YL, Lu KT. Neonatal glucocorticoid treatment increased depression-like behaviour in adult rats. International Journal of Neuropsychopharmacology. 2014((Ko M.-C.; Hung Y.-H.; Lu K.-T.) Department of Life Science, National Taiwan Normal University, Taipei, Taiwan).  125. Koletzko B, Boey CCM, Campoy C, Carlson SE, Chang N, Guillermo-Tuazon MA, et al. Current information and asian perspectives on long-chain polyunsaturated fatty acids in pregnancy, lactation, and infancy: Systematic review and practice recommendations from an early nutrition academy workshop. Annals of Nutrition and Metabolism. 2014;65(1):49-80.  126. Koos BJ, Rajaee A. Fetal breathing movements and changes at birth. 2014. p. 89-101.  127. Mouzi L, Ashutosh W. Anesthetic management during labor and delivery of a multiparous patient terminally ill with metastatic breast cancer. A and A Case Reports. 2014;2(4):48-9.  128. Palmsten K, Huybrechts KF, Kowal MK, Mogun H, Hernández-Díaz S. Validity of maternal and infant outcomes within nationwide Medicaid data. Pharmacoepidemiology and Drug Safety. 2014;23(6):646-55.  129. Pimentel VM, Eckardt MJ. More than interpreters needed: The specialized care of the immigrant pregnant patient. Obstetrical and Gynecological Survey. 2014;69(8):490-500.  130. Ray S, Stowe ZN. The use of antidepressant medication in pregnancy. Best Practice and Research: Clinical Obstetrics and Gynaecology. 2014;28(1):71-83.  131. Rodríguez-Martínez CE, Sossa-Briceño MP, Castro-Rodriguez JA. Predictors of hospitalization for asthma in children: Results of a 1-year prospective study. Pediatric Pulmonology. 2014;49(11):1058-64.  132. Saiyed R, Rand CM, Carroll MS, Weese-Mayer DE. Hypoventilation syndromes of infancy, childhood, and adulthood: Congenital central hypoventilation syndrome (CCHS), later-onset CCHS, and rapid-onset obesity with hypothalamic dysfunction, hypoventilation, and autonomic dysregulation. Sleep Medicine Clinics. 2014;9(3):425-39.  133. Salmanian B, Shamshirsaz AA, Cass DL, Javadian P, Ruano R, Ayres NA, et al. Fetal cardiac tamponade in a case of right-side congenital diaphragmatic hernia. Obstetrics and Gynecology. 2014;123(SUPPL. 2):447-50.  134. Teoh WHL, Westphal M, Kampmeier TG. Update on volume therapy in obstetrics. Best Practice and Research: Clinical Anaesthesiology. 2014;28(3):297-303.  135. Acar G, Simsek Z, Avci A, Aung SM, Koca F, Saglam M, et al. Right heart free-floating thrombus in a pregnant woman with massive pulmonary embolism: A case of 'emboli in transit'. Journal of Cardiovascular Medicine. 2015;16((Acar G., doctorgokselacar@hotmail.com; Simsek Z.; Avci A.; Aung S.M.; Koca F.; Saglam M.; Kaymaz C.) Department of Cardiology, Kartal Kosuyolu Heart and Research Hospital, Denizer Cd. Cevizli Kavşaǧi No: 2 Cevizli, Kartal, Istanbul, Turkey):S51-S4.  136. Anoshiravani A, Saynina O, Chamberlain L, Goldstein BA, Huffman LC, Wang NE, et al. Mental Illness Drives Hospitalizations for Detained California Youth. Journal of Adolescent Health. 2015;57(5):455-61.  137. Bellet F, Beyens MN, Bernard N, Beghin D, Elefant E, Vial T. Exposure to aripiprazole during embryogenesis: A prospective multicenter cohort study. Pharmacoepidemiology and Drug Safety. 2015;24(4):368-80.  138. Bordbar A, Farjadnia M. Maternal Morbidities and Occurrence of Intraventricular Hemorrhage in Preterm Infants. Journal of Pediatric Infectious Diseases. 2015;1(3):156-61.  139. Casper RC. Use of selective serotonin reuptake inhibitor antidepressants in pregnancy does carry risks, but the risks are small. Journal of Nervous and Mental Disease. 2015;203(3):167-9.  140. Cheng TS, Chen H, Lee T, Teoh OH, Shek LP, Lee BW, et al. An independent association of prenatal depression with wheezing and anxiety with rhinitis in infancy. Pediatric Allergy and Immunology. 2015;26(8):765-71.  141. Domingues MR, Bassani DG, da Silva SG, de Vargas Nunes Coll C, da Silva BGC, Hallal PC. Physical activity during pregnancy and maternal-child health (PAMELA): Study protocol for a randomized controlled trial. Trials. 2015;16(1).  142. Du Toit E, Thomas E, Koen L, Vythilingum B, Grobler S, Smith N, et al. SSRI use in pregnancy: Evaluating the risks and benefits. South African Journal of Psychiatry. 2015;21(2):48-53.  143. Emmett PM, Jones LR, Golding J. Pregnancy diet and associated outcomes in the Avon Longitudinal Study of Parents and Children. Nutrition Reviews. 2015;73((Emmett P.M., p.m.emmett@bristol.ac.uk; Golding J.) Centre for Child and Adolescent Health, School of Social and Community Medicine, University of Bristol, Bristol, United Kingdom):154-74.  144. Ferro MA, Boyle MH, Alati R, Scott JG, Dingle K. Maternal psychological distress mediates the relationship between asthma and physician visits in a population-based sample of adolescents. Journal of Asthma. 2015;52(2):170-5.  145. Ferro MA, Boyle MH, Avison WR. Association between trajectories of maternal depression and subsequent psychological functioning in youth with and without chronic physical illness. Health Psychology. 2015;34(8):820-8.  146. Grantz KL, Hinkle SN, Mendola P, Sjaarda LA, Leishear K, Albert PS. Differences in Risk Factors for Recurrent Versus Incident Preterm Delivery. American Journal of Epidemiology. 2015;182(2):157-67.  147. Groer MW, Gregory KE, Louis-Jacques A, Thibeau S, Walker WA. The very low birth weight infant microbiome and childhood health. Birth Defects Research Part C - Embryo Today: Reviews. 2015;105(4):252-64.  148. Huybrechts KF, Bateman BT, Palmsten K, Desai RJ, Patorno E, Gopalakrishnan C, et al. Antidepressant use late in pregnancy and risk of persistent pulmonary hypertension of the newborn. JAMA - Journal of the American Medical Association. 2015;313(21):2142-51.  149. Li Y, Jiang Y, Li S, Shen X, Liu J, Jiang F. Pre-and postnatal risk factors in relation to allergic rhinitis in school-aged children in China. PLoS ONE. 2015;10(2).  150. Mei-Dan E, Ray JG, Vigod SN. Perinatal outcomes among women with bipolar disorder: A population-based cohort study. American Journal of Obstetrics and Gynecology. 2015;212(3):367.e1-.e8.  151. Mirani G, Williams PL, Chernoff M, Abzug MJ, Levin MJ, Seage GR, et al. Changing Trends in Complications and Mortality Rates among US Youth and Young Adults with HIV Infection in the Era of Combination Antiretroviral Therapy. Clinical Infectious Diseases. 2015;61(12):1850-61.  152. Newton ER, Hale TW. Drugs in breast milk. Clinical Obstetrics and Gynecology. 2015;58(4):868-84.  153. Noskova P, Blaha J, Bakhouche H, Kubatova J, Ulrichova J, Marusicova P, et al. Neonatal effect of remifentanil in general anaesthesia for caesarean section: A randomized trial. BMC Anesthesiology. 2015;15(1).  154. Pearlstein T. Depression during Pregnancy. Best Practice and Research: Clinical Obstetrics and Gynaecology. 2015;29(5):754-64.  155. Rabie NZ, Bird TM, Magann EF, Hall RW, McKelvey SS. ADHD and developmental speech/language disorders in late preterm, early term and term infants. Journal of Perinatology. 2015;35(8):660-4.  156. Robinson GE. Controversies about the use of antidepressants in pregnancy. Journal of Nervous and Mental Disease. 2015;203(3):159-63.  157. Roth R, Lynch K, Lernmark B, Baxter J, Simell T, Smith L, et al. Maternal anxiety about a child's diabetes risk in the TEDDY study: The potential role of life stress, postpartum depression, and risk perception. Pediatric Diabetes. 2015;16(4):287-98.  158. Saito-Benz M, Miller HE, Berry MJ. Shwachman-Diamond syndrome (SDS) in a preterm neonate. Journal of Paediatrics and Child Health. 2015;51(12):1228-31.  159. Størksen HT, Garthus-Niegel S, Adams SS, Vangen S, Eberhard-Gran M. Fear of childbirth and elective caesarean section: A population-based study. BMC Pregnancy and Childbirth. 2015;15(1).  160. Yaari M, Millo I, Harel A, Friedlander E, Bar-Oz B, Eventov-Friedman S, et al. Predicting maternal resolution of preterm birth at one month corrected age. Infancy. 2015;20(5):507-22.  161. Al Sayed Mohamed H, Al Jaber MM, Al-Hamadani Z, Khmour HY, Al Lenjawi BA, Schlogl JM. Prevalence of postnatal depression and associated risk factors among south asian mothers living in a newly developing country. Asian Journal of Pharmaceutical and Clinical Research. 2016;9(6):57-61.  162. Allen VM, Baskett TF, Allen AC, Burrows J, Vincer M, O'Connell CM. Type of Labour in the First Pregnancy and Cumulative Perinatal Morbidity. Journal of Obstetrics and Gynaecology Canada. 2016;38(9):804-10.  163. Barthow C, Wickens K, Stanley T, Mitchell EA, Maude R, Abels P, et al. The Probiotics in Pregnancy Study (PiP Study): Rationale and design of a double-blind randomised controlled trial to improve maternal health during pregnancy and prevent infant eczema and allergy. BMC Pregnancy and Childbirth. 2016;16(1).  164. Bérard A, Zhao JP, Sheehy O. Success of smoking cessation interventions during pregnancy. American Journal of Obstetrics and Gynecology. 2016;215(5):611.e1-.e8.  165. Chang HY, Suh DI, Yang SI, Kang MJ, Lee SY, Lee E, et al. Prenatal maternal distress affects atopic dermatitis in offspring mediated by oxidative stress. Journal of Allergy and Clinical Immunology. 2016;138(2):468-75.e5.  166. Chung EK, Siegel BS, Garg A, Conroy K, Gross RS, Long DA, et al. Screening for Social Determinants of Health among Children and Families Living in Poverty: A Guide for Clinicians. Current Problems in Pediatric and Adolescent Health Care. 2016;46(5):135-53.  167. Coburn SS, Gonzales NA, Luecken LJ, Crnic KA. Multiple domains of stress predict postpartum depressive symptoms in low-income Mexican American women: the moderating effect of social support. Archives of Women's Mental Health. 2016;19(6):1009-18.  168. Coleman AM, Merrow AC, Crombleholme TM, Jaekle R, Lim FY. Fetal MRI of Torsed Bronchopulmonary Sequestration with Tension Hydrothorax and Hydrops in a Twin Gestation. Fetal Diagnosis and Therapy. 2016;40(2):156-60.  169. Cucchi D, Menon A, Feroldi FM, Kwapisz A, Randelli F, Cabitza F, et al. Risk factors for post-operative shoulder stiffness: Are there new candidates? Journal of Biological Regulators and Homeostatic Agents. 2016;30(4):123-9.  170. Devkota R, Khan GM, Alam K, Regmi A, Sapkota B. Medication utilization pattern for management of pregnancy complications: A study in Western Nepal. BMC Pregnancy and Childbirth. 2016;16(1).  171. Ferro MA, Van Lieshout RJ, Ohayon J, Scott JG. Emotional and behavioral problems in adolescents and young adults with food allergy. Allergy: European Journal of Allergy and Clinical Immunology. 2016;71(4):532-40.  172. Ferro MA, Van Lieshout RJ, Scott JG, Alati R, Mamun AA, Dingle K. Condition-specific associations of symptoms of depression and anxiety in adolescents and young adults with asthma and food allergy. Journal of Asthma. 2016;53(3):282-8.  173. Habek D, Dujaković T, Habek JC, Jurković I. Twenty-three-year long-term health outcome after the war in Vukovar. Acta Clinica Croatica. 2016;55(1):58-62.  174. Jølving LR, Nielsen J, Kesmodel US, Nielsen RG, Beck-Nielsen SS, Nørgård BM. Prevalence of maternal chronic diseases during pregnancy – a nationwide population based study from 1989 to 2013. Acta Obstetricia et Gynecologica Scandinavica. 2016;95(11):1295-304.  175. Lazenby GB, Mmeje O, Fisher BM, Weinberg A, Aaron EK, Keating M, et al. Antiretroviral resistance and pregnancy characteristics of women with perinatal and nonperinatal HIV Infection. Infectious Diseases in Obstetrics and Gynecology. 2016;2016((Lazenby G.B., lazenbgb@musc.edu) Department of Obstetrics and Gynecology, Medical University of South Carolina, 96 Jonathan Lucas Street, Charleston, SC, United States).  176. Li W, Li H, Long Y. Clinical Characteristics and Long-term Predictors of Persistent Left Ventricular Systolic Dysfunction in Peripartum Cardiomyopathy. Canadian Journal of Cardiology. 2016;32(3):362-8.  177. Nevels RM, Gontkovsky ST, Williams BE. Paroxetine—the antidepressant from hell? Probably not, but caution required. Psychopharmacology Bulletin. 2016;46(1):77-104.  178. Pace CC, Spittle AJ, Molesworth CML, Lee KJ, Northam EA, Cheong JLY, et al. Evolution of depression and anxiety symptoms in parents of very preterm infants during the newborn period. JAMA Pediatrics. 2016;170(9):863-70.  179. Petersen I, Evans SJ, Gilbert R, Marston L, Nazareth I. Selective serotonin reuptake inhibitors and congenital heart anomalies: Comparative cohort studies of women treated before and during pregnancy and their children. Journal of Clinical Psychiatry. 2016;77(1):e36-e42.  180. Rosenthal JL, Hilton JF, Teufel RJ, Romano PS, Kaiser SV, Okumura MJ. Profiling interfacility transfers for hospitalized pediatric patients. Hospital Pediatrics. 2016;6(6):345-53.  181. Sharkey KM, Iko IN, Machan JT, Thompson-Westra J, Pearlstein TB. Infant sleep and feeding patterns are associated with maternal sleep, stress, and depressed mood in women with a history of major depressive disorder (MDD). Archives of Women's Mental Health. 2016;19(2):209-18.  182. Singal D, Brownell M, Chateau D, Ruth C, Katz LY. Neonatal and childhood neurodevelopmental, health and educational outcomes of children exposed to antidepressants and maternal depression during pregnancy: Protocol for a retrospective population-based cohort study using linked administrative data. BMJ Open. 2016;6(11).  183. Teng JY, Yin Ing Chee C, Chong YS, Lee LY, Yong EL, Chi C, et al. A suicidal pregnant patient's request for premature Cesarean section: Clinical and ethical challenges. Journal of Affective Disorders. 2016;194((Teng J.Y., jia_ying_teng@nuhs.edu.sg; Yin Ing Chee C.) Department of Psychological Medicine, National University Hospital, Singapore):168-70.  184. Vivilaki VG, Diamanti A, Tzeli M, Patelarou E, Bick D, Papadakis S, et al. Exposure to active and passive smoking among Greek pregnant women. Tobacco Induced Diseases. 2016;14(1).  185. Wang IJ, Wen HJ, Chiang TL, Lin SJ, Guo YL. Maternal psychologic problems increased the risk of childhood atopic dermatitis. Pediatric Allergy and Immunology. 2016;27(2):169-76.  186. Askew K, Bamford J, Hudson N, Moratelli J, Miller R, Anderson A, et al. Current characteristics, challenges and coping strategies of young people with cystic fibrosis as they transition to adulthood. Clinical Medicine, Journal of the Royal College of Physicians of London. 2017;17(2):121-5.  187. Bérard A, Sheehy O, Zhao JP, Vinet É, Bernatsky S, Abrahamowicz M. SSRI and SNRI use during pregnancy and the risk of persistent pulmonary hypertension of the newborn. British Journal of Clinical Pharmacology. 2017;83(5):1126-33.  188. Berry JG, Ash AS, Cohen E, Hasan F, Feudtner C, Hall M. Contributions of children with multiple chronic conditions to pediatric hospitalizations in the United States: A retrospective cohort analysis. Hospital Pediatrics. 2017;7(7):365-72.  189. Braig S, Weiss JM, Stalder T, Kirschbaum C, Rothenbacher D, Genuneit J. Maternal prenatal stress and child atopic dermatitis up to age 2 years: The Ulm SPATZ health study. Pediatric Allergy and Immunology. 2017;28(2):144-51.  190. Brzenski A, Greenberg M. Neonatal abstinence syndrome due to in-utero exposure to SSRI: A case report. Internet Journal of Pediatrics and Neonatology. 2017;19(1).  191. Chiu YHM, Sheffield PE, Hsu HHL, Goldstein J, Curtin PC, Wright RJ. Subconstructs of the Edinburgh Postnatal Depression Scale in a multi-ethnic inner-city population in the U.S. Archives of Women's Mental Health. 2017;20(6):803-10.  192. Dodd JM, Crowther CA, Grivell RM, Deussen AR. Elective repeat caesarean section versus induction of labour for women with a previous caesarean birth. Cochrane Database of Systematic Reviews. 2017;2017(7):1-13.  193. Dorsey MJ, Dvorak CC, Cowan MJ, Puck JM. Treatment of infants identified as having severe combined immunodeficiency by means of newborn screening. Journal of Allergy and Clinical Immunology. 2017;139(3):733-42.  194. Dubovicky M, Belovicova K, Csatlosova K, Bogi E. Risks of using SSRI / SNRI antidepressants during pregnancy and lactation. Interdisciplinary Toxicology. 2017;10(1):30-4.  195. Elbert NJ, Duijts L, den Dekker HT, de Jong NW, Nijsten TEC, Jaddoe VWV, et al. Maternal psychiatric symptoms during pregnancy and risk of childhood atopic diseases. Clinical and Experimental Allergy. 2017;47(4):509-19.  196. El-Heis S, Crozier SR, Healy E, Robinson SM, Harvey NC, Cooper C, et al. Maternal stress and psychological distress preconception: association with offspring atopic eczema at age 12 months. Clinical and Experimental Allergy. 2017;47(6):760-9.  197. Fernandez S. You can reduce secondhand smoke exposure! prescribing nicotine replacement in the pediatrician’s office. Pediatric Annals. 2017;46(9):e315-e8.  198. Górniaczyk A, Czech-Szczapa B, Sobkowski M, Chmaj-Wierzchowska K. Maternal health-related behaviours during pregnancy: a critical public health issue. European Journal of Contraception and Reproductive Health Care. 2017;22(4):321-5.  199. Karlsson L, Nousiainen N, Scheinin NM, Maksimow M, Salmi M, Lehto SM, et al. Cytokine profile and maternal depression and anxiety symptoms in mid-pregnancy—the FinnBrain Birth Cohort Study. Archives of Women's Mental Health. 2017;20(1):39-48.  200. Kim CH, Kim SH, Lee JS. Association of maternal depression and allergic diseases in Korean children. Allergy and Asthma Proceedings. 2017;38(4):300-8.  201. Letourneau NL, Kozyrskyj AL, Cosic N, Ntanda HN, Anis L, Hart MJ, et al. Maternal sensitivity and social support protect against childhood atopic dermatitis. Allergy, Asthma and Clinical Immunology. 2017;13(1).  202. Mamyrbayeva MA, Zhumagaliyeva GD, Altynnik NA, Dmitrashchenko AA. The risk of intrauterine infections in newborns in Aktobe Region of Kazakhstan. Asian Journal of Pharmaceutics. 2017;11(1):S136-S45.  203. Murphy VE, Jensen ME, Gibson PG. Asthma during Pregnancy: Exacerbations, Management, and Health Outcomes for Mother and Infant. Seminars in Respiratory and Critical Care Medicine. 2017;38(2):160-73.  204. Özdoğan Ş, Kurtaraner T, Gencer H, Kabakcı-Kaya D, Çelik-Erden S. Association between maternal depression and wheezing in preschool children. Turkish Journal of Pediatrics. 2017;58(6):632-40.  205. Rendtorff R, Hinkson L, Kiver V, Dröge LA, Henrich W. Pregnancies in Women Aged 45 Years and Older - a 10-Year Retrospective Analysis in Berlin. Geburtshilfe und Frauenheilkunde. 2017;77(3):268-75.  206. Sahrakorpi N, Koivusalo SB, Stach-Lempinen B, Eriksson JG, Kautiainen H, Roine RP. “The burden of pregnancy”; heavier for the heaviest? The changes in health related quality of life (HRQoL) assessed by the 15D instrument during pregnancy and postpartum in different body mass index groups: A longitudinal survey. Acta Obstetricia et Gynecologica Scandinavica. 2017;96(3):352-8.  207. Scott RK, Chakhtoura N, Burke MM, Cohen RA, Kreitchmann R. Delivery after 40 Weeks of Gestation in Pregnant Women with Well-Controlled Human Immunodeficiency Virus. Obstetrics and Gynecology. 2017;130(3):502-10.  208. Sharma JB, Kriplani A, Sharma E, Sharma S, Dharmendra S, Kumar S, et al. Multi drug resistant female genital tuberculosis: A preliminary report. European Journal of Obstetrics and Gynecology and Reproductive Biology. 2017;210((Sharma J.B., jbsharma2000@gmail.com; Kriplani A.; Sharma E.; Dharmendra S.; Kumar S.) Department of Obstetrics and Gynaecology, All India Institute of Medical Sciences, New Delhi, India):108-15.  209. Tattoli L, Di Vella G, Solarino B. A case of intrauterine lethal fetal injury after attempted suicide of the mother. Forensic Science International. 2017;280((Tattoli L., luciatattoli@libero.it; Di Vella G., giancarlo.divella@unito.it) Department of Public Health and Pediatrics, Section of Legal Medicine, University of Turin, Corso Galileo Galilei 22, Torino, Italy):e1-e5.  210. Trindade MC, Bittencourt T, Lorenzi-Filho G, Alves RC, de Andrade DC, Fonoff ET, et al. Restless legs syndrome in Wilson's disease: frequency, characteristics, and mimics. Acta Neurologica Scandinavica. 2017;135(2):211-8.  211. Truong BT, Lupattelli A, Kristensen P, Nordeng H. Sick leave and medication use in pregnancy: A European web-based study. BMJ Open. 2017;7(8).  212. Voltas N, Arija V, Hernández-Martínez C, Jiménez-Feijoo R, Ferré N, Canals J. Are there early inflammatory biomarkers that affect neurodevelopment in infancy? Journal of Neuroimmunology. 2017;305((Voltas N.; Arija V.; Hernández-Martínez C.; Canals J., josefa.canals@urv.cat) Research Center for Behavioral Assessment (CRAMC), Department of Psychology, Universitat Rovira i Virgili, Facultat de Ciències de l'Educació i Psicologia, Crta/ de Valls s/n, Tarragona, Spain):42-50.  213. Abrams EJ, Mellins CA, Bucek A, Dolezal C, Raymond J, Wiznia A, et al. Behavioral health and adult milestones in young adults with perinatal HIV infection or exposure. Pediatrics. 2018;142(3).  214. Aurora S, Aurora N, Datta P, Rewers-Felkins K, Baker T, Hale TW. Evaluating transfer of modafinil into human milk during lactation: A case report. Journal of Clinical Sleep Medicine. 2018;14(12):2087-9.  215. Barnfield L, Neale E, Reynolds S. Outpatient cervical ripening in a district general hospital: a five-year retrospective cohort study. Journal of Obstetrics and Gynaecology. 2018;38(3):301-4.  216. Bowers K, Ding L, Gregory S, Yolton K, Ji H, Meyer J, et al. Maternal distress and hair cortisol in pregnancy among women with elevated adverse childhood experiences. Psychoneuroendocrinology. 2018;95((Bowers K., Katherine.bowers@cchmc.org; Ding L.; Gregory S.; Folger A.) Cincinnati Children's Hospital Medical Center, Division of Biostatistics and Epidemiology, Cincinnati OH, 3333 Burnet Ave, Cincinnati, OH, United States):145-8.  217. Brew BK, Lundholm C, Viktorin A, Lichtenstein P, Larsson H, Almqvist C. Longitudinal depression or anxiety in mothers and offspring asthma: A Swedish populationbased study. International Journal of Epidemiology. 2018;47(1):166-74.  218. Buffa G, Dahan S, Sinclair I, St-Pierre M, Roofigari N, Mutran D, et al. Prenatal stress and child development: A scoping review of research in low- and middle-income countries. PLoS ONE. 2018;13(12).  219. Calvo-Ferrandiz E, Peraita-Adrados R. Narcolepsy with cataplexy and pregnancy: a case–control study. Journal of Sleep Research. 2018;27(2):268-72.  220. Collin-Lévesque L, El-Ghaddaf Y, Genest M, Jutras M, Leclair G, Weisskopf E, et al. Infant Exposure to Methylphenidate and Duloxetine during Lactation. Breastfeeding Medicine. 2018;13(3):221-5.  221. Denny JT, Rocke ZM, McRae VA, Denny JE, Fratzola CH, Ibrar S, et al. Varicella Pneumonia: Case Report and Review of a Potentially Lethal Complication of a Common Disease. Journal of Investigative Medicine High Impact Case Reports. 2018;6((Denny J.T., dennyjt@rwjms.rutgers.edu; McRae V.A.; Fratzola C.H.; Ibrar S.; Bonitz J.; Tse J.T.; Cohen S.; Mellender S.J.; Kiss G.K.) Rutgers University, New Brunswick, NJ, United States).  222. Dos Santos F, Drymiotou S, Antequera Martin A, Mol BW, Gale C, Devane D, et al. Development of a core outcome set for trials on induction of labour: an international multistakeholder Delphi study. BJOG: An International Journal of Obstetrics and Gynaecology. 2018;125(13):1673-80.  223. Goh DA, Gan D, Kung J, Baron-Cohen S, Allison C, Chen H, et al. Child, Maternal and Demographic Factors Influencing Caregiver-Reported Autistic Trait Symptomatology in Toddlers. Journal of Autism and Developmental Disorders. 2018;48(4):1325-37.  224. Gottlieb M, Long B, Koyfman A. Approach to the Agitated Emergency Department Patient. Journal of Emergency Medicine. 2018;54(4):447-57.  225. Hamajima E, Noda M, Nai E, Akiyama S, Ikuta Y, Obana N, et al. Therapy with propylthiouracil for T3-predominant neonatal Graves’ disease: A case report. Clinical Pediatric Endocrinology. 2018;27(3):171-8.  226. Kang LJ, Koleva PT, Field CJ, Giesbrecht GF, Wine E, Becker AB, et al. Maternal depressive symptoms linked to reduced fecal Immunoglobulin A concentrations in infants. Brain, Behavior, and Immunity. 2018;68((Kang L.J.; Koleva P.T.; Kozyrskyj A.L., kozyrsky@ualberta.ca) Department of Pediatrics, University of Alberta, 3-527 Edmonton Clinic Health Academy, 11405–87 Avenue, Edmonton, Alberta, Canada):123-31.  227. Keepanasseril A, Maurya DK, Manikandan K, Suriya Y, Habeebullah S, Raghavan SS. Prophylactic magnesium sulphate in prevention of eclampsia in women with severe preeclampsia: randomised controlled trial (PIPES trial). Journal of Obstetrics and Gynaecology. 2018;38(3):305-9.  228. Langhammer K, Roth B, Kribs A, Göpel W, Kuntz L, Miedaner F. Treatment and outcome data of very low birth weight infants treated with less invasive surfactant administration in comparison to intubation and mechanical ventilation in the clinical setting of a cross-sectional observational multicenter study. European Journal of Pediatrics. 2018;177(8):1207-17.  229. Litvin DG, Dick TE, Smith CB, Jacono FJ. Lung-injury depresses glutamatergic synaptic transmission in the nucleus tractus solitarii via discrete age-dependent mechanisms in neonatal rats. Brain, Behavior, and Immunity. 2018;70((Litvin D.G.; Smith C.B.) Department of Physiology & Biophysics, Case Western Reserve University School of Medicine, Cleveland, OH, United States):398-422.  230. Liu JM, Chiu FH, Liu YP, Chen SP, Chan HH, Yang JJ, et al. Antepartum urinary tract infection and postpartum depression in Taiwan - a nationwide population-based study. BMC Pregnancy and Childbirth. 2018;18(1).  231. Ozturk Z, Kalayci CC. Pregnancy outcomes in psychiatric patients treated with passiflora incarnata. Complementary Therapies in Medicine. 2018;36((Ozturk Z., dr.zeyneb@hotmail.com) Department of Clinical Pharmacology and Toxicology, Izmir Ataturk Research Hospital, Izmir, Turkey):30-2.  232. Perera FP, Wheelock K, Wang Y, Tang D, Margolis AE, Badia G, et al. Combined effects of prenatal exposure to polycyclic aromatic hydrocarbons and material hardship on child ADHD behavior problems. Environmental Research. 2018;160((Perera F.P., fpp1@columbia.edu; Wheelock K., kmw2189@cumc.columbia.edu; Tang D., dt14@cumc.columbia.edu; Cowell W., wc2449@cumc.columbia.edu; Miller R.L., rlm14@cumc.columbia.edu; Herbstman J.B., jh2678@cumc.columbia.edu) Department of Environmental Health Sciences, Mailman School of Public Health, Columbia University, 722W. 168th Street, New York, NY, United States):506-13.  233. Popovic M, Pizzi C, Rusconi F, Gagliardi L, Galassi C, Trevisan M, et al. The role of maternal anorexia nervosa and bulimia nervosa before and during pregnancy in early childhood wheezing: Findings from the NINFEA birth cohort study. International Journal of Eating Disorders. 2018;51(8):842-51.  234. Slykerman RF, Kang J, Van Zyl N, Barthow C, Wickens K, Stanley T, et al. Effect of early probiotic supplementation on childhood cognition, behaviour and mood a randomised, placebo-controlled trial. Acta Paediatrica, International Journal of Paediatrics. 2018;107(12):2172-8.  235. Symington EA, Baumgartner J, Malan L, Zandberg L, Ricci C, Smuts CM. Nutrition during pregnancy and early development (NuPED) in urban South Africa: A study protocol for a prospective cohort. BMC Pregnancy and Childbirth. 2018;18(1).  236. Vilchez G, Dai J, Kumar K, Mundy D, Kontopoulos E, Sokol RJ. Racial/ethnic disparities in magnesium sulfate neuroprotection: a subgroup analysis of a multicenter randomized controlled trial. Journal of Maternal-Fetal and Neonatal Medicine. 2018;31(17):2304-11.  237. Andrade C. Intellectual disability after gestational exposure to antidepressant drugs: The confidence interval as a compatibility interval. Journal of Clinical Psychiatry. 2019;80(3).  238. Badell ML, Sheth AN, Momplaisir F, Rahangdale L, Potter J, Woodham PC, et al. A multicenter analysis of elvitegravir use during pregnancy on HIV viral suppression and perinatal outcomes. Open Forum Infectious Diseases. 2019;6(4).  239. Cardenas A, Faleschini S, Cortes Hidalgo A, Rifas-Shiman SL, Baccarelli AA, Demeo DL, et al. Prenatal maternal antidepressants, anxiety, and depression and offspring DNA methylation: Epigenome-wide associations at birth and persistence into early childhood. Clinical Epigenetics. 2019;11(1).  240. Crane DA, Doody DR, Schiff MA, Mueller BA. Pregnancy Outcomes in Women with Spinal Cord Injuries: A Population-Based Study. PM and R. 2019;11(8):795-806.  241. Grand K, Gonzalez-Gandolfi C, Ackermann AM, Aljeaid D, Bedoukian E, Bird LM, et al. Hyperinsulinemic hypoglycemia in seven patients with de novo NSD1 mutations. American Journal of Medical Genetics, Part A. 2019;179(4):542-51.  242. Gumbie M, Parkinson B, Cutler H, Gauld N, Mumford V. Is Reclassification of the Oral Contraceptive Pill from Prescription to Pharmacist-Only Cost Effective? Application of an Economic Evaluation Approach to Regulatory Decisions. PharmacoEconomics. 2019;37(8):1049-64.  243. Güngör Ş, Kırık S, Özkars MY, Korulmaz A. Effect of maternal depression and environmental factors on infantile colic. Erciyes Medical Journal. 2019;41(1):80-4.  244. Hahn J, Gold DR, Coull BA, McCormick MC, Finn PW, Perkins DL, et al. Prenatal Maternal Depression and Neonatal Immune Responses. Psychosomatic Medicine. 2019;81(4):320-7.  245. Hamann CR, Egeberg A, Silverberg JI, Gislason G, Skov L, Thyssen JP. Exploring the association between parental psychiatric disease and childhood atopic dermatitis: a matched case–control study. Journal of the European Academy of Dermatology and Venereology. 2019;33(4):725-34.  246. Kasparian NA, Kan JM, Sood E, Wray J, Pincus HA, Newburger JW. Mental health care for parents of babies with congenital heart disease during intensive care unit admission: Systematic review and statement of best practice. Early Human Development. 2019;139((Kasparian N.A., nadine.kasparian@childrens.harvard.edu; Newburger J.W.) Department of Pediatrics, Harvard Medical School, Boston, MA, United States).  247. Korhonen LS, Kortesluoma S, Lukkarinen M, Peltola V, Pesonen H, Pelto J, et al. Prenatal maternal distress associates with a blunted cortisol response in rhinovirus-positive infants. Psychoneuroendocrinology. 2019;107((Korhonen L.S., lasula@utu.fi; Kortesluoma S.; Lukkarinen M.; Peltola V.; Pesonen H.; Pelto J.; Tuulari J.J.; Lukkarinen H.; Karlsson H.; Karlsson L.) FinnBrain Birth Cohort Study, Turku Brain and Mind Center, Department of Clinical Medicine, University of Turku, Lemminkäisenkatu 3A, Teutori building, 2(nd)floor, Turku, Finland):187-90.  248. Kozhimannil KB, Interrante JD, Henning-Smith C, Admon LK. Rural-urban differences in severe maternal morbidity and mortality in the us, 2007–15. Health Affairs. 2019;38(12):2077-85.  249. Lemal R, Fouquet G, Terriou L, Vaes M, Livideanu CB, Frenzel L, et al. Omalizumab Therapy for Mast Cell-Mediator Symptoms in Patients with ISM, CM, MMAS, and MCAS. Journal of Allergy and Clinical Immunology: In Practice. 2019;7(7):2387-95.e3.  250. Liu X, Helenius D, Skotte L, Beaumont RN, Wielscher M, Geller F, et al. Variants in the fetal genome near pro-inflammatory cytokine genes on 2q13 associate with gestational duration. Nature Communications. 2019;10(1).  251. Magee LA, De Silva DA, Sawchuck D, Synnes A, von Dadelszen P. No. 376-Magnesium Sulphate for Fetal Neuroprotection. Journal of Obstetrics and Gynaecology Canada. 2019;41(4):505-22.  252. Mascarenhas MR. Pediatric anti-inflammatory diet. Pediatric Annals. 2019;48(6):e220-e5.  253. Mnisi B, Makin J, Lindeque BG, Adam S. Postnatal depressive features in mothers of neonates admitted to a neonatal unit at steve biko academic hospital: The role of sociodemographic and psychosocial factors. South African Journal of Obstetrics and Gynaecology. 2019;25(3):89-94.  254. Murthy P, Clark D. An unusual cause for neonatal abstinence syndrome. Paediatrics and Child Health (Canada). 2019;24(1):12-4.  255. Ng QX, Venkatanarayanan N, Ho CYX, Sim WS, Lim DY, Yeo WS. Selective Serotonin Reuptake Inhibitors and Persistent Pulmonary Hypertension of the Newborn: An Update Meta-Analysis. Journal of Women's Health. 2019;28(3):331-8.  256. Oh DJ, Chen JL, Vajaranant TS, Dikopf MS. Brimonidine tartrate for the treatment of glaucoma. Expert Opinion on Pharmacotherapy. 2019;20(1):115-22.  257. Rochtus AM, Trowbridge S, Goldstein RD, Sheidley BR, Prabhu SP, Haynes R, et al. Mutations in NRXN1 and NRXN2 in a patient with early-onset epileptic encephalopathy and respiratory depression. Cold Spring Harbor Molecular Case Studies. 2019;5(1).  258. Skajaa N, Szépligeti SK, Xue F, Sørensen HT, Ehrenstein V, Eisele O, et al. Pregnancy, Birth, Neonatal, and Postnatal Neurological Outcomes After Pregnancy With Migraine. Headache. 2019;59(6):869-79.  259. Sun Y, Pedersen LH, Wu CS, Petersen I, Sørensen HT, Olsen J. Antidepressant use during pregnancy and risk of congenital heart defects: A case-time-control study. Pharmacoepidemiology and Drug Safety. 2019;28(9):1180-93.  260. Tetuan C, Axon DR, Bingham J, Boesen K, Lipsy R, Scovis N, et al. Assessing the effect of a telepharmacist's recommendations during an integrated, interprofessional telehealth appointment and their alignment with quality measures. Journal of Managed Care and Specialty Pharmacy. 2019;25(12):1334-9A.  261. Weinstein SL. The Natural History of Adolescent Idiopathic Scoliosis. Journal of Pediatric Orthopaedics. 2019;39(6):S44-S6.  262. Xiao J, Fogarty C, Wu TT, Alkhers N, Zeng Y, Thomas M, et al. Oral health and candida carriage in socioeconomically disadvantaged us pregnant women. BMC Pregnancy and Childbirth. 2019;19(1).  263. Yadav V, Sharma JB, Kachhawa G, Kulshrestha V, Mahey R, Kumari R, et al. Obstetrical and perinatal outcome in pregnant women with extrapulmonary tuberculosis. Indian Journal of Tuberculosis. 2019;66(1):158-62.  264. Yolcu M, Bilal MS, Avsar MK, Yildirim O. A patient with anomalous origin of the left coronary artery from the pulmonary artery (ALCAPA syndrome) and 13 live births. Cardiovascular Journal of Africa. 2019;30(3):E1-E2.  265. Zenhäusern J, Bekker A, Wates MA, Schaaf HS, Dramowski A. Tuberculosis transmission in a hospitalised neonate: Need for optimised tuberculosis screening of pregnant and postpartum women. South African Medical Journal. 2019;109(5):310-3.  266. Andersen JT, Futtrup TB. Drugs during lactation. Adverse Drug Reaction Bulletin. 2020;323(1):1251-4.  267. Anderson KN, Lind JN, Simeone RM, Bobo WV, Mitchell AA, Riehle-Colarusso T, et al. Maternal Use of Specific Antidepressant Medications during Early Pregnancy and the Risk of Selected Birth Defects. JAMA Psychiatry. 2020;77(12):1246-55.  268. Bauer D, Tüchler R, Dörfler D, Lawitschka A. Nature’s endless wonder: unexpected motherhood after pediatric allogeneic stem cell transplantation and severe late effects. Wiener Klinische Wochenschrift. 2020;132(7-8):210-4.  269. Beex-Oosterhuis MM, Samb A, Heerdink ER, Souverein PC, Van Gool AR, Meyboom RHB, et al. Safety of clozapine use during pregnancy: Analysis of international pharmacovigilance data. Pharmacoepidemiology and Drug Safety. 2020;29(6):725-35.  270. Brito S, Sampaio I, Dinis A, Proença E, Vilan A, Soares E, et al. Use of therapeutic hypothermia in sudden unexpected postnatal collapse: A retrospective study. Acta Medica Portuguesa. 2020;34(13).  271. Castro-Rodriguez JA, Forno E, Casanello P, Padilla O, Krause BJ, Uauy R. Leptin in cord blood associates with asthma risk at age 3 in the offspring of women with gestational obesity. Annals of the American Thoracic Society. 2020;17(12):1583-9.  272. Chung EK, Short VL, Hand DJ, Gubernick RS, Abatemarco DJ. Poor prenatal care does not predict well child care for children born to mothers with opioid use disorder. Journal of Substance Use. 2020;25(5):482-8.  273. Gagliano A, Galati C, Ingrassia M, Ciuffo M, Alquino MA, Tanca MG, et al. Pediatric acute-onset neuropsychiatric syndrome: A data mining approach to a very specific constellation of clinical variables. Journal of Child and Adolescent Psychopharmacology. 2020;30(8):495-511.  274. Ge GM, Leung MTY, Man KKC, Leung WC, Ip P, Li GHY, et al. Maternal thyroid dysfunction during pregnancy and the risk of adverse outcomes in the offspring: A systematic review and meta-analysis. Journal of Clinical Endocrinology and Metabolism. 2020;105(12).  275. Hasegawa Y, Miyata M, Miura S, Nagata A, Tomonaga C, Shigetomi N, et al. Genetic counseling for trisomy x syndrome diagnosed by amniocentesis: A case report. Acta Medica Nagasakiensia. 2020;64(1):31-4.  276. Ibrahim A, Hussain N. Brief report: Metabolic acidosis in newborn infants following maternal use of acetazolamide during pregnancy. Journal of Neonatal-Perinatal Medicine. 2020;13(3):419-25.  277. İnkaya AC, Örgül G, Halis N, Alp Ş, Kara A, Özyüncü Ö, et al. Perinatal outcomes of twenty-five human immunodeficiency virus-infected pregnant women: Hacettepe university experience. Journal of the Turkish German Gynecology Association. 2020;21(3):180-6.  278. Jaddoe VWV, Felix JF, Andersen AMN, Charles MA, Chatzi L, Corpeleijn E, et al. The LifeCycle Project-EU Child Cohort Network: a federated analysis infrastructure and harmonized data of more than 250,000 children and parents. European Journal of Epidemiology. 2020;35(7):709-24.  279. Kang LJ, Vu KN, Koleva PT, Field CJ, Chow A, Azad MB, et al. Maternal psychological distress before birth influences gut immunity in mid-infancy. Clinical and Experimental Allergy. 2020;50(2):178-88.  280. Kapnadak SG, Dimango E, Hadjiliadis D, Hempstead SE, Tallarico E, Pilewski JM, et al. Cystic Fibrosis Foundation consensus guidelines for the care of individuals with advanced cystic fibrosis lung disease. Journal of Cystic Fibrosis. 2020;19(3):344-54.  281. Kariuki SM, Gray DM, Newton CRJC, Vanker A, MacGinty RP, Koen N, et al. Association between maternal psychological adversity and lung function in South African infants: A birth cohort study. Pediatric Pulmonology. 2020;55(1):236-44.  282. Kim JH, Kim JY, Lee J, Jeong GH, Lee E, Lee S, et al. Environmental risk factors, protective factors, and peripheral biomarkers for ADHD: an umbrella review. The Lancet Psychiatry. 2020;7(11):955-70.  283. Kompaniyets L, Lundeen EA, Belay B, Goodman AB, Tangka F, Blanck HM. Hospital Length of Stay, Charges, and Costs Associated with a Diagnosis of Obesity in US Children and Youth, 2006-2016. Medical Care. 2020;58(8):722-6.  284. Lameijer H, Schutte JM, Schuitemaker NWE, van Roosmalen JJM, Pieper PG. Maternal mortality due to cardiovascular disease in the Netherlands: a 21-year experience. Netherlands Heart Journal. 2020;28(1):27-36.  285. Li HH. Hereditary angioedema: Long-term prophylactic treatment. Allergy and Asthma Proceedings. 2020;41((Li H.H., hl@allergyasthma.us) Institute for Asthma and Allergy, PC, 2 Wisconsin Circle, Suite 250, Chevy Chase, MD, United States):S35-S7.  286. Maia GN, Frizzo GB, Levandowski DC. Psychofunctional symptoms in infants of young mothers: Association with maternal mental health and parental bonding. Early Human Development. 2020;141((Maia G.N., gabisnmaia@gmail.com) Graduate Program in Health Sciences, Federal University of Health Sciences of Porto Alegre, Porto Alegre, RS, Brazil).  287. Matera MG, Page CP, Calzetta L, Rogliani P, Cazzola M. Pharmacology and therapeutics of bronchodilators revisited. Pharmacological Reviews. 2020;72(1):218-52.  288. McKenzie C, Silverberg JI. Maternal Depression and Atopic Dermatitis in American Children and Adolescents. Dermatitis. 2020;31(1):75-80.  289. Mehler K, Hucklenbruch-Rother E, Trautmann-Villalba P, Becker I, Roth B, Kribs A. Delivery room skin-to-skin contact for preterm infants—A randomized clinical trial. Acta Paediatrica, International Journal of Paediatrics. 2020;109(3):518-26.  290. Menter A, Gelfand JM, Connor C, Armstrong AW, Cordoro KM, Davis DMR, et al. Joint American Academy of Dermatology–National Psoriasis Foundation guidelines of care for the management of psoriasis with systemic nonbiologic therapies. Journal of the American Academy of Dermatology. 2020;82(6):1445-86.  291. Milano W, Ambrosio P, Carizzone F, De Biasio V, Capasso A. Obesity: Diagnosis and treatment. Pharmacologyonline. 2020;3((Milano W., wamilano@tin.it; Ambrosio P.; Carizzone F.; De Biasio V.) UOSD Eating Disorder Unit, Mental Health Department ASL Napoli 2 Nord, Napoli, Italy):89-93.  292. Moreno-Encinas A, Sepúlveda AR, Kurland V, Lacruz T, Nova E, Graell M. Identifying psychosocial and familial correlates and the impact of the stressful life events in the onset of anorexia nervosa: Control-case study (ANOBAS): Psychosocial and familial correlates and stressful life events in AN. Psychiatry Research. 2020;284((Moreno-Encinas A., alba.moreno@uam.es; Sepúlveda A.R.; Kurland V.; Lacruz T.) School of Psychology. Autonomous University of Madrid, Spain).  293. Oliva M, Hsu K, Alsamarai S, Chavez VD, Ferrara L. Clinical improvement of severe COVID-19 pneumonia in a pregnant patient after caesarean delivery. BMJ Case Reports. 2020;13(7).  294. Rabiei S, Hajian P, Pirdehghan A, Mabodi K, Khansari S. Comparison of the effects of epidural and spinal anesthesia on analgesia and blood gases in neonates born by natural vaginal delivery: A clinical trial study. Biomedical Research and Therapy. 2020;7(3):3686-92.  295. Ramani A, Testa G, Ghouri Y, Koon EC, Di Salvo M, McKenna GJ, et al. DUETS (Dallas UtErus Transplant Study): Complete report of 6-month and initial 2-year outcomes following open donor hysterectomy. Clinical Transplantation. 2020;34(1).  296. Razavi B, Kasraeian M, Hashemi A, Alamdarloo SM, Najib FS. Complex twisted knots of umbilical cord in a monochorionic-diamniotic twin gestation: A case report. Galen Medical Journal. 2020;9((Razavi B.; Hashemi A., atefehashemi68@gmail.com; Alamdarloo S.M.; Najib F.S.) Department of Obstetrics and Gynecology, Shiraz University of Medical Sciences, Shiraz, Iran).  297. Robijn AL, Brew BK, Jensen ME, Rejnö G, Lundholm C, Murphy VE, et al. Effect of maternal asthma exacerbations on perinatal outcomes: A population-based study. ERJ Open Research. 2020;6(4).  298. Royal C, Gray C. Allergy prevention: An overview of current evidence. Yale Journal of Biology and Medicine. 2020;93(5):689-98.  299. Schauberger CW, Borgert AJ, Bearwald B. Continuation in Treatment and Maintenance of Custody of Newborns After Delivery in Women With Opioid Use Disorder. Journal of Addiction Medicine. 2020;14(2):119-25.  300. Sorensen CJ, Salas RN, Rublee C, Hill K, Bartlett ES, Charlton P, et al. Clinical Implications of Climate Change on US Emergency Medicine: Challenges and Opportunities. Annals of Emergency Medicine. 2020;76(2):168-78.  301. Stefanovic V. COVID-19 infection during pregnancy: Fetus as a patient deserves more attention. Journal of Perinatal Medicine. 2020;48(5):438-40.  302. Suresh R, Fatima S, Ratnani I, Masud F, Reardon MJ. Emergent Transcatheter Aortic Valve Replacement (TAVR) Performed on Patient With DiGeorge Syndrome. Cardiovascular Revascularization Medicine. 2020;21(11):36-8.  303. Vari D, Xiao W, Behere S, Spurrier E, Tsuda T, Baffa JM. Low-dose prostaglandin E1 is safe and effective for critical congenital heart disease: is it time to revisit the dosing guidelines? Cardiology in the Young. 2020((Vari D.) Department of Pediatrics, Nemours/Alfred I. duPont Hospital for Children, Wilmington, DE, United States).  304. Wei D, Au Yeung SL, Lu M, Xiao W, Lu J, Shen S, et al. Association between prenatal depressive symptoms and eczema in infants: The Born in Guangzhou Cohort Study. Pediatric Allergy and Immunology. 2020;31(6):662-70.  305. Whalen OM, Campbell LE, Murphy VE, Lane AE, Gibson PG, Mattes J, et al. Observational study of mental health in asthmatic women during the prenatal and postnatal periods. Journal of Asthma. 2020;57(8):829-41.  306. Ahmad K, Kabir E, Keramat SA, Khanam R. Maternal health and health-related behaviours and their associations with child health: Evidence from an Australian birth cohort. PLoS ONE. 2021;16(9 September).  307. Aigbokhaode AQ, Isara AR. Household air pollution and respiratory symptoms of women and children in a suburban community in nigeria. Turkish Thoracic Journal. 2021;22(6):466-72.  308. Badmanaban R, Saha D, Sen DJ, Biswas A, Mandal S, Basak S. Turmeric: A holistic solution for biochemical malfunction. Research Journal of Pharmacy and Technology. 2021;14(10):5540-50.  309. Bokern MP, Robijn AL, Jensen ME, Barker D, Callaway L, Clifton V, et al. Factors Associated with Asthma Exacerbations During Pregnancy. Journal of Allergy and Clinical Immunology: In Practice. 2021;9(12):4343-52.e4.  310. Bowers K, Ding L, Yolton K, Ji H, Nidey N, Meyer J, et al. Pregnancy and Infant Development (PRIDE)—a preliminary observational study of maternal adversity and infant development. BMC Pediatrics. 2021;21(1).  311. Braun WE, Herlitz L, Li J, Schold J, Poggio E, Stephany B, et al. Continuous function of 80 primary renal allografts for 30–47 years with maintenance prednisone and azathioprine/mycophenolate mofetil therapy: A clinical mosaic of long-term successes. Clinical Transplantation. 2021;35(1).  312. Camerota M, Graw S, Everson TM, McGowan EC, Hofheimer JA, O’Shea TM, et al. Prenatal risk factors and neonatal DNA methylation in very preterm infants. Clinical Epigenetics. 2021;13(1).  313. Chen S. Are prenatal anxiety or depression symptoms associated with asthma or atopic diseases throughout the offspring’s childhood? An updated systematic review and meta-analysis. BMC Pregnancy and Childbirth. 2021;21(1).  314. Chighizola CB, Crisafulli F, Hoxha A, Carubbi F, Bellan M, Monti S, et al. Psychosocial burden in young patients with primary anti-phospholipid syndrome: An Italian nationwide survey (the AQUEOUS study). Clinical and Experimental Rheumatology. 2021;39(5):938-46.  315. Cornish RP, MacLeod J, Boyd A, Tilling K. Factors associated with participation over time in the Avon Longitudinal Study of Parents and Children: A study using linked education and primary care data. International Journal of Epidemiology. 2021;50(1):293-302.  316. Cutroneo PM, Isgrò V, Ientile V, Santarpia M, Ferlazzo G, Fontana A, et al. Safety profile of immune checkpoint inhibitors: An analysis of the Italian spontaneous reporting system database. British Journal of Clinical Pharmacology. 2021;87(2):527-41.  317. Dagher RK, Bruckheim HE, Colpe LJ, Edwards E, White DB. Perinatal Depression: Challenges and Opportunities. Journal of Women's Health. 2021;30(2):154-9.  318. Damsky W, Peterson D, Ramseier J, Al-Bawardy B, Chun H, Proctor D, et al. The emerging role of Janus kinase inhibitors in the treatment of autoimmune and inflammatory diseases. Journal of Allergy and Clinical Immunology. 2021;147(3):814-26.  319. Dubrall D, Leitzen S, Toni I, Stingl J, Schulz M, Schmid M, et al. Descriptive analysis of adverse drug reaction reports in children and adolescents from Germany: frequently reported reactions and suspected drugs. BMC Pharmacology and Toxicology. 2021;22(1).  320. Dundon KMW, Powell WT, Wilder JL, King B, Schwartz A, McPhillips H, et al. Parenthood and parental leave decisions in pediatric residency. Pediatrics. 2021;148(4).  321. Gillespie ML, Nemastil CJ, Moore-Clingenpeel M, Gilmore D, Dell ML, Krivchenia K. Mental health history and social barriers impacting caregivers of infants with cystic fibrosis. Pediatric Pulmonology. 2021;56(2):457-64.  322. Hachulla E, Agard C, Allanore Y, Avouac J, Bader-Meunier B, Belot A, et al. French recommendations for the management of systemic sclerosis. Orphanet Journal of Rare Diseases. 2021;16((Hachulla E., eric.hachulla@chru-lille.fr; Condette-Wojtasik G.; Launay D.; Sobanski V.) Service de Médecine Interne et Immunologie Clinique, Centre de Référence Des Maladies Autoimmunes Systémiques Rares du Nord et Nord-Ouest de France (CeRAINO), Univ. Lille, Inserm, CHU Lille, U1286 - INFINITE - Institute for Translational Research in Inflammation, Lille, France).  323. Hahn J, Gold DR, Coull BA, McCormick MC, Finn PW, Perkins DL, et al. Air pollution, neonatal immune responses, and potential joint effects of maternal depression. International Journal of Environmental Research and Public Health. 2021;18(10).  324. Han VX, Patel S, Jones HF, Nielsen TC, Mohammad SS, Hofer MJ, et al. Maternal acute and chronic inflammation in pregnancy is associated with common neurodevelopmental disorders: a systematic review. Translational Psychiatry. 2021;11(1).  325. He C, Xiao G, Liu S, Hua Z, Wang L, Wang N. A prospective cohort study of cord blood 25(OH)D3 and food allergies in 6-month-old Chinese infants. Asian Pacific Journal of Allergy and Immunology. 2021;39(4):258-65.  326. Howley MM, Werler MM, Fisher SC, Van Zutphen AR, Carmichael SL, Broussard CS, et al. Maternal exposure to hydroxychloroquine and birth defects. Birth Defects Research. 2021;113(17):1245-56.  327. Ilic M, Nordeng H, Lupattelli A. Medical care contact for infertility and related medication use during pregnancy – a european, cross-sectional web-based study. Norsk Epidemiologi. 2021;29(1-2):97-106.  328. Jürges H, Kopetsch T. Prenatal exposure to the German food crisis 1944–1948 and health after 65 years. Economics and Human Biology. 2021;40((Jürges H., juerges@uni-wuppertal.de) University of Wuppertal, MEA, DIW, and ROA, Rainer-Gruenter-Str. 21 (FN), Wuppertal, Germany).  329. Kim BV, Aromataris EC, Middleton P, Townsend R, Thangaratinam S, Duffy JMN, et al. Development of a core outcome set for interventions to prevent stillbirth. Australian and New Zealand Journal of Obstetrics and Gynaecology. 2021;61(5):658-66.  330. Leone M, Kuja-Halkola R, Leval A, D'Onofrio BM, Larsson H, Lichtenstein P, et al. Association of Youth Depression with Subsequent Somatic Diseases and Premature Death. JAMA Psychiatry. 2021;78(3):302-10.  331. Lisanti AJ, Demianczyk AC, Vaughan K, Martino GF, Ohrenschall RS, Quinn R, et al. Parental role alteration strongly influences depressive symptoms in mothers of preoperative infants with congenital heart disease. Heart and Lung. 2021;50(2):235-41.  332. Mahesh S, Mallappa M, Habchi O, Konstanta V, Chise C, Sykiotou P, et al. Appearance of Acute Inflammatory State Indicates Improvement in Atopic Dermatitis Cases Under Classical Homeopathic Treatment: A Case Series. Clinical Medicine Insights: Case Reports. 2021;14((Mahesh S., bhatseema@hotmail.com) School of Medicine, Faculty of Health and Medical Sciences, Taylor’s University, Subang Jaya, Malaysia).  333. Mehler K, Broer A, Roll C, Göpel W, Wieg C, Jahn P, et al. Developmental outcome of extremely preterm infants is improved after less invasive surfactant application: Developmental outcome after LISA. Acta Paediatrica, International Journal of Paediatrics. 2021;110(3):818-25.  334. Mohapatra B, Sarangi I. Women and sexual and reproductive health rights: Issues and challenges due to environmental pollution and covid-19 pandemic. Indian Journal of Forensic Medicine and Toxicology. 2021;15(4):127-33.  335. Ramratnam SK, Lockhart A, Visness CM, Calatroni A, Jackson DJ, Gergen PJ, et al. Maternal stress and depression are associated with respiratory phenotypes in urban children. Journal of Allergy and Clinical Immunology. 2021;148(1):120-7.  336. Ren L, Chen Q, Min S, Peng F, Wang B, Yu J, et al. Labor Analgesia reduces the risk of postpartum depression: A cohort study. Translational Neuroscience. 2021;12(1):396-406.  337. Ren T, Chen J, Yu Y, He H, Zhang J, Li F, et al. The association of asthma, atopic dermatitis, and allergic rhinitis with peripartum mental disorders. Clinical and Translational Allergy. 2021;11(10).  338. Ruohomäki A, Toffol E, Airaksinen V, Backman K, Voutilainen R, Hantunen S, et al. The impact of postpartum depressive symptoms on self-reported infant health and analgesic consumption at the age of 12 months: A prospective cohort study. Journal of Psychiatric Research. 2021;136((Ruohomäki A., ruohomaki.aleksi@gmail.com; Airaksinen V.; Lehto S.M.) Institute of Clinical Medicine / Psychiatry, University of Eastern Finland, P.O. Box 1627, Kuopio, FI, Finland):388-97.  339. Ryan LM, Mahmood DMA, Laurence PCO. Incidence of concomitant illnesses in pregnancy in Indonesia: Estimates from 1990–2019, with projections to 2030. The Lancet Regional Health - Western Pacific. 2021;10((Ryan L.M., lareesa.ryan@adelaide.edu.au; Mahmood D.M.A.; Laurence P.C.O.) School of Public Health, University of Adelaide, South Australia, Australia).  340. Saito J, Ishii M, Mito A, Yakuwa N, Kawasaki H, Tachibana Y, et al. Trazodone Levels in Maternal Serum, Cord Blood, Breast Milk, and Neonatal Serum. Breastfeeding Medicine. 2021;16(11):922-5.  341. Saito J, Ishii M, Miura Y, Yakuwa N, Kawasaki H, Suzuki T, et al. Brotizolam during Pregnancy and Lactation: Brotizolam Levels in Maternal Serum, Cord Blood, Breast Milk, and Neonatal Serum. Breastfeeding Medicine. 2021;16(7):579-82.  342. Singh A, Kaur H, Gupta G, Naranje K, Verma A, Roy A, et al. Enhancement of Immunity and Health in Neonates and Infants. Journal of Neonatology. 2021;35(3):138-54.  343. Spaeth AM, Khetarpal R, Yu D, Pien GW, Herring SJ. Determinants of postpartum sleep duration and sleep efficiency in minority women. Sleep. 2021;44(4).  344. Stålberg V, Josefsson A, Bladh M, Lilliecreutz C. The risk of postpartum hemorrhage when lowering the oxytocin dose in planned cesarean section, a pilot study. Sexual and Reproductive Healthcare. 2021;29((Stålberg V.) Department of Obstetrics and Gynecology in Norrköping and Division of Children's and Women's Health, Department of Biomedical and Clinical Sciences, Faculty of Medicine and Health Sciences, Linköping University, Sweden).  345. Stevens EL, Han YY, Rosser F, Forno E, Acosta-Pérez E, Miller GE, et al. Maternal Depressive Symptoms, Lung Function, and Severe Asthma Exacerbations in Puerto Rican Children. Journal of Allergy and Clinical Immunology: In Practice. 2021;9(3):1319-26.e3.  346. Walker S, Dasgupta T, Halliday A, Reitter A. Development of a core outcome set for effectiveness studies of breech birth at term (Breech-COS): A systematic review on variations in outcome reporting. European Journal of Obstetrics and Gynecology and Reproductive Biology. 2021;263((Walker S., Shawn.Walker@kcl.ac.uk; Dasgupta T., Tisha.Dasgupta@kcl.ac.uk; Halliday A.) King's College London, Department of Women and Children's Health, London, United Kingdom):117-26.  347. Wang S, Wei Y, Liu L, Li Z. Association Between Breastmilk Microbiota and Food Allergy in Infants. Frontiers in Cellular and Infection Microbiology. 2021;11((Wang S.; Li Z., topbj163@sina.com) Department of Pediatrics, Peking University Third Hospital, Beijing, China).  348. Yamamoto-Hanada K, Pak K, Saito-Abe M, Sato M, Ohya Y. Better maternal quality of life in pregnancy yields better offspring respiratory outcomes: A birth cohort. Annals of Allergy, Asthma and Immunology. 2021;126(6):713-21.e1.  349. Yilmaz O, Yasar A, Caliskan Polat A, Ay P, Alkin T, Taneli F, et al. Maternal psychiatric status and infant wheezing: The role of maternal hormones and cord blood cytokines. Pediatric Pulmonology. 2021;56(6):1573-82.  350. Zejnullahu VA, Ukella-Lleshi D, Miftari E, Govori V. Prevalence of postpartum depression at the clinic for obstetrics and gynecology in Kosovo teaching hospital: Demographic, obstetric and psychosocial risk factors. European Journal of Obstetrics and Gynecology and Reproductive Biology. 2021;256((Zejnullahu V.A.; Ukella-Lleshi D.; Miftari E.) Department of Obstetrics and Gynecology, University Clinical Center of Kosovo, Pristina, Kosovo, Albania):215-20.  351. Zhu H, Wu J, Yang Y, Li X, Hu R. Risk of Neonatal Short-Term Adverse Outcomes Associated with Noninfectious Intrapartum Hyperthermia: A Nested Case-Control Retrospective Study. American Journal of Perinatology. 2021;38(5):507-14.  352. Zou ML, Jiang CB, Chen YH, Wu CD, Candice Lung SC, Chien LC, et al. Effects of air pollution, land-use type, and maternal mental health on child development in the first two years of life in the Greater Taipei area. Environmental Research. 2021;197((Zou M.-L.; Chen Y.-H.; Chien L.-C.; Yang Y.-T.; Chao H.J., hchao@tmu.edu.tw) School of Public Health, College of Public Health, Taipei Medical University, Taipei, Taiwan).  353. Adkins EA, Yolton K, Strawn JR, Lippert F, Ryan PH, Brunst KJ. Fluoride exposure during early adolescence and its association with internalizing symptoms. Environmental Research. 2022;204((Adkins E.A.; Brunst K.J., kelly.brunst@uc.edu) Department of Environmental and Public Health Sciences, University of Cincinnati, 160 Panzeca Way, Cincinnati, OH, United States).  354. Aker AM, Stephenson AL, Wilton AS, Vigod SN, Dennis CL, Guttmann A, et al. Asthma Severity and Control and Their Association With Perinatal Mental Illness. Canadian Journal of Psychiatry. 2022;67(2):156-9.  355. Aker AM, Vigod SN, Dennis CL, Brown HK. Perinatal Complications as a Mediator of the Association Between Chronic Disease and Postpartum Mental Illness. Journal of Women's Health. 2022;31(4):564-72.  356. Aker AM, Vigod SN, Dennis CL, Kaster T, Brown HK. The association between asthma and perinatal mental illness: a population-based cohort study. International Journal of Epidemiology. 2022;51(3):964-73.  357. Aleem S, Walker LS, Hornik CD, Smith MJ, Grotegut CA, Weimer KED. Severe Congenital Syphilis in the Neonatal Intensive Care Unit: A Retrospective Case Series. Pediatric Infectious Disease Journal. 2022;41(4):335-9.  358. Alinaghi SAS, Karimi A, Pashaei Z, Afzalian A, PegahMirzapour, Ghorbanzadeh K, et al. Safety and Adverse Events Related to COVID-19 mRNA Vaccines; a Systematic Review. Archives of Academic Emergency Medicine. 2022;10(1).  359. Bagagiolo D, Debora R, Borrelli F. Efficacy and safety of osteopathic manipulative treatment: An overview of systematic reviews. BMJ Open. 2022;12(4).  360. Batmaz SB, Birinci G, Aslan EA. Quality of life of children with allergic disease: the effect of depression and anxiety of children and their mothers. Journal of Asthma. 2022;59(9):1776-86.  361. Binder C, Schmid P, Abele H, Graf J. Does Antenatal MgSO4 Administration to the Mother in the Event of Imminent Premature Birth Reduce the Occurrence of Infantile Cerebral Palsy in the Child? – An Umbrella Review. Geburtshilfe und Frauenheilkunde. 2022;83(5):602-11.  362. Brännström M, Dahm-Kähler P, Kvarnström N, Enskog A, Olofsson JI, Olausson M, et al. Reproductive, obstetric, and long-term health outcome after uterus transplantation: results of the first clinical trial. Fertility and Sterility. 2022;118(3):576-85.  363. Buffoni I, Buratti S, Mallamaci MF, Pezzato S, Lampugnani E, Buffelli F, et al. Sudden Onset of Severe Pulmonary Hypertension in a Preterm Infant: A Case Report on the Role of Maternal Use of Serotonin Re-Uptake Inhibitors During Pregnancy and Concurrent Risk Factors. Frontiers in Pediatrics. 2022;10((Buffoni I., isabella.buffoni@gmail.com; Buratti S.; Mallamaci M.F.; Pezzato S.; Lampugnani E.; Moscatelli A.) Division of Neonatal and Pediatric Intensive Care, Emergency Department, IRCCS Giannina Gaslini Institute, Genoa, Italy).  364. Catalao R, Dorrington S, Pritchard M, Jewell A, Broadbent M, Ashworth M, et al. Ethnic inequalities in mental and physical multimorbidity in women of reproductive age: a data linkage cohort study. BMJ Open. 2022;12(7).  365. Clemente-Teixeira M, Magalhães T, Barrocas J, Dinis-Oliveira RJ, Taveira-Gomes T. Health Outcomes in Women Victims of Intimate Partner Violence: A 20-Year Real-World Study. International Journal of Environmental Research and Public Health. 2022;19(24).  366. Coté JJ, Granger P, Mishra A, Sorini G. COVID-19 in a pregnant cystic fibrosis carrier with myasthenia gravis: A case report. Case Reports in Women's Health. 2022;34((Coté J.J., john.cote@commonspirit.org; Granger P.; Mishra A.) Obstetrics and Gynecology, Creighton University School of Medicine, Omaha, NE, United States).  367. Deierlein AL, Litvak J, Stein CR. Preconception Health and Disability Status Among Women of Reproductive Age Participating in the National Health and Nutrition Examination Surveys, 2013-2018. Journal of Women's Health. 2022;31(9):1320-33.  368. Dennis CL, Prioreschi A, Brown HK, Brennenstuhl S, Bell RC, Atkinson S, et al. Medical, behavioural and social preconception and interconception risk factors among pregnancy planning and recently pregnant Canadian women. Family Medicine and Community Health. 2022;10(3).  369. Dumitru AE, Gică C, Iordăchescu DA, Panaitescu AM, Peltecu G, Botezatu R, et al. GESTATIONAL SURROGACY. MEDICAL, PSYCHOLOGICAL AND LEGAL ASPECTS. Romanian Journal of Legal Medicine. 2022;29(3):323-7.  370. Dür M, Röschel A, Oberleitner-Leeb C, Herrmanns V, Pichler-Stachl E, Mattner B, et al. Associations Between Parental Occupational Balance, Subjective Health, and Clinical Characteristics of VLBW Infants. Frontiers in Pediatrics. 2022;10((Dür M., mona.duer@duervation.com; Röschel A.) Department of Health Sciences, IMC University of Applied Sciences Krems, Krems, Austria).  371. Eleftheriades M, Vousoura E, Eleftheriades A, Pervanidou P, Zervas IM, Chrousos G, et al. Physical Health, Media Use, Stress, and Mental Health in Pregnant Women during the COVID-19 Pandemic. Diagnostics. 2022;12(5).  372. Fujioka I, Ohtsu H, Yonemoto N, Sase K, Murashima A. Association between prenatal exposure to antidepressants and neonatal morbidity: An analysis of real-world data from a nationwide claims database in Japan. Journal of Affective Disorders. 2022;310((Fujioka I., fujioka-i@ncchd.go.jp; Murashima A.) The Japan Drug Information Institute in Pregnancy, National Center for Child Health and Development, Japan):60-7.  373. Galletta MAK, de Oliveira Demo M, Pinto ALT, Francisco RPV, Zugaib M. Demographic, Clinical, and Obstetrical Characterization of Women with Hyperemesis Gravidarum Using a More Restrictive Diagnosis. Clinical and Experimental Obstetrics and Gynecology. 2022;49(10).  374. Gangbe EM, Badeghiesh A, Baghlaf H, Dahan MH. Pregnancy, delivery, and neonatal outcomes among women with psoriatic arthritis, a population based study. Journal of Perinatal Medicine. 2022;50(5):581-6.  375. Goicochea Ríos EDS, Córdova Paz Soldán OM, Gómez Goicochea NI, Vicuña Villacorta J. POST-INFECTION SEQUELAE OF COVID 19 AMONG PATIENTS OF HOSPITAL I.FLORENCIA DE MORA. Trujillo - Peru. Revista de la Facultad de Medicina Humana. 2022;22(4):754-64.  376. Guille C, Johnson E, Douglas E, Aujla R, Boyars L, Kruis R, et al. A Pilot Study Examining Access to and Satisfaction with Maternal Mental Health and Substance Use Disorder Treatment via Telemedicine. Telemedicine Reports. 2022;3(1):24-9.  377. Hashimoto Y, Yamana H, Michihata N, Shigemi D, Ishimaru M, Matsui H, et al. Eye drops for dry eye disease during pregnancy and adverse neonatal outcomes: high-dimensional propensity score analyses. Ophthalmic Epidemiology. 2022;29(4):384-93.  378. Havdahl A, Wootton RE, Leppert B, Riglin L, Ask H, Tesli M, et al. Associations between Pregnancy-Related Predisposing Factors for Offspring Neurodevelopmental Conditions and Parental Genetic Liability to Attention-Deficit/Hyperactivity Disorder, Autism, and Schizophrenia: The Norwegian Mother, Father and Child Cohort Study (MoBa). JAMA Psychiatry. 2022;79(8):799-810.  379. Haxel CS, Johnson JN, Hintz S, Renno MS, Ruano R, Zyblewski SC, et al. Care of the Fetus With Congenital Cardiovascular Disease: From Diagnosis to Delivery. Pediatrics. 2022;150((Haxel C.S.) The University of Vermont Children's Hospital, Burlington, VT, United States).  380. Heinonen E, Forsberg L, Nörby U, Wide K, Källén K. Neonatal morbidity after fetal exposure to antipsychotics: a national register-based study. BMJ Open. 2022;12(6).  381. Hemavathi P, Malathi S. Polycystic Ovary Syndromeand Risk of Polycystic Ovary Syndrome among Adolescent Girls in selected Colleges, Puducherry. Cardiometry. 2022;24((Hemavathi P., breezehema@gmail.com; Malathi S.) Department of Community Health Nursing, Vinayaka Missions Annapoorana College of Nursing, Vinayaka Mission’s Research Foundation (Deemed to be University), Salem, India):987-92.  382. Hung KH, Tsao SL, Yang SF, Wang BY, Huang JY, Li WT, et al. Association of General Anesthesia and Neuraxial Anesthesia in Caesarean Section with Maternal Postpartum Depression: A Retrospective Nationwide Population-Based Cohort Study. Journal of Personalized Medicine. 2022;12(6).  383. Jeong K, Kim J, Chang HY, Song TW, Kim JH, Shin M, et al. Maternal Posttraumatic Stress Symptoms and Psychological Burden in Mothers of Korean Children with Anaphylaxis. Allergy, Asthma and Immunology Research. 2022;14(6):742-51.  384. Kan A, Ture M, Yilmaz K, Emre E, Bas G. Maternal Anxiety, Stress, and Depression: The Role of Food Allergy. Asthma Allergy Immunology. 2022;20(2):95-100.  385. Keil C, Bedei I, Sommer L, Koemhoff M, Axt-Fliedner R, Köhler S, et al. Fetal therapy of LUTO (lower urinary tract obstruction)–a follow-up observational study. Journal of Maternal-Fetal and Neonatal Medicine. 2022;35(25):8536-43.  386. Kojima R, Shinohara R, Kushima M, Horiuchi S, Otawa S, Yokomichi H, et al. Prenatal Negative Life Events and Childhood Allergies: The Japan Environment and Children's Study (JECS). International Archives of Allergy and Immunology. 2022;183(10):1062-70.  387. Lau HX, Kee MZL, Yap QV, Tham EH, Chan YH, Goh AEN, et al. Associations Between Maternal Distress During Early Life Periods and Offspring Respiratory Infections and Allergic Outcomes. Frontiers in Pediatrics. 2022;10((Lau H.X.; Kee M.Z.L.; Tham E.H.; Eriksson J.G.; Gluckman P.D.; Chong Y.S.; Meaney M.J.; Loo E.X.L., evelyn_loo@sics.a-star.edu.sg) Singapore Institute for Clinical Sciences (SICS), Agency for Science, Technology and Research (A*STAR), Singapore, Singapore).  388. Lee SI, Azcoaga-Lorenzo A, Agrawal U, Kennedy JI, Fagbamigbe AF, Hope H, et al. Epidemiology of pre-existing multimorbidity in pregnant women in the UK in 2018: a population-based cross-sectional study. BMC Pregnancy and Childbirth. 2022;22(1).  389. Lewinn KZ, Karr CJ, Hazlehurst M, Carroll K, Loftus C, Nguyen R, et al. Cohort profile: The ECHO prenatal and early childhood pathways to health consortium (ECHO-PATHWAYS). BMJ Open. 2022;12(10).  390. Logue TC, Wen T, Monk C, Guglielminotti J, Huang Y, Wright JD, et al. Trends in and complications associated with mental health condition diagnoses during delivery hospitalizations. American Journal of Obstetrics and Gynecology. 2022;226(3):405.e1-.e16.  391. Lundholm C, Rejnö G, Brew B, Smew AI, Saltvedt S, Almqvist C. Associations Between Maternal Distress, Cortisol Levels, and Perinatal Outcomes. Psychosomatic Medicine. 2022;84(3):288-96.  392. Mallise CA, Murphy VE, Karayanidis F, Armstrong H, Whalen OM, Woolard AJ, et al. Parenting stress in mothers with asthma during the postpartum period. Journal of Asthma. 2022;59(10):2091-9.  393. Martini J, Asselmann E, Weidner K, Knappe S, Rosendahl J, Garthus-Niegel S. Prospective Associations of Lifetime Post-traumatic Stress Disorder and Birth-Related Traumatization With Maternal and Infant Outcomes. Frontiers in Psychiatry. 2022;13((Martini J., julia.martini@tu-dresden.de) Department of Psychiatry and Psychotherapy, Faculty of Medicine of the Technische Universität Dresden, Dresden, Germany).  394. Mehdi MQ, Franco Fuenmayor ME, Aly AM. Discordant expression of maternal SLE in twin pregnancy with a single fetal AV block: A case report. Journal of Neonatal-Perinatal Medicine. 2022;15(4):863-6.  395. Morales Torres J, Aceves FJ, Amigo Castañeda MC, Hernández Cuevas CB. Could Frida Kahlo have had antiphospholipid syndrome? Reumatologia Clinica. 2022;18(2):65-8.  396. Roach V. Perinatal depression and anxiety. Medicine Today. 2022;23(8):72-4.  397. Rodriguez N, Tessier CA, Mandhane PJ, Pei J, Simons E, Moraes TJ, et al. Sex-specific associations among infant food and atopic sensitizations and infant neurodevelopment. Frontiers in Pediatrics. 2022;10((Rodriguez N.; Tessier C.A.; Mandhane P.J.; Kozyrskyj A.L., kozyrsky@ualberta.ca) Department of Pediatrics, Faculty of Medicine and Dentistry, University of Alberta, Edmonton, AB, Canada).  398. Rommel AS, Momen NC, Molenaar NM, Agerbo E, Bergink V, Munk-Olsen T, et al. Antidepressant use during pregnancy and risk of adverse neonatal outcomes: A comprehensive investigation of previously identified associations. Acta Psychiatrica Scandinavica. 2022;145(6):544-56.  399. Sakya SM, Hallan DR, Maczuga SA, Kirby JS. Outcomes of pregnancy and childbirth in women with hidradenitis suppurativa. Journal of the American Academy of Dermatology. 2022;86(1):61-7.  400. Sanseverino PB, Hoffmann A, Machado S, Farias M, Michels MS, Sanseverino MTV, et al. High-risk twin pregnancy: case report of an adolescent patient with cystic fibrosis and systemic lupus erythematosus. Journal of Medical Case Reports. 2022;16(1).  401. Shrivastava A, Bansal A, Amar S. PREVALENCE AND MANAGEMENT OF POSTPARTUM ANAEMIA: A TERTIARY CARE TEACHING HOSPITAL BASED STUDY. International Journal of Academic Medicine and Pharmacy. 2022;4(5):416-9.  402. Spencer JP, Thomas S, Trondsen Pawlowski RH. Medication Safety in Breastfeeding. American Family Physician. 2022;106(6):638-44.  403. Suarez EA, Haug N, Hansbury A, Stojanovic D, Corey C. Prescription medication use and baseline health status of women with live-birth deliveries in a national data network. American Journal of Obstetrics and Gynecology MFM. 2022;4(1).  404. Trinh TG, Schwarze CE, Müller M, Goetz M, Hassdenteufel K, Wallwiener M, et al. Implementing a Perinatal Depression Screening in Clinical Routine: Exploring the Patient s Perspective. Geburtshilfe und Frauenheilkunde. 2022;82(10):1082-92.  405. Uccellini O, Benlodi A, Caroppo E, Cena L, Esposito G, Fernandez I, et al. 1000 Days: The “WeCare Generation” Program—The Ultimate Model for Improving Human Mental Health and Economics: The Study Protocol. International Journal of Environmental Research and Public Health. 2022;19(24).  406. Vinekar A, Sinha S, Mohan A, Mangalesh S, Jayadev C, Shetty B. Role of Birth Order in the Development of Retinopathy of Prematurity in Multiple Gestation: a Study of 1040 Asian Indian Premature Infants. SN Comprehensive Clinical Medicine. 2022;4(1).  407. Wang S, Zhang R, Li X, Gao Y, Dai N, Wei Y, et al. Relationship between maternal–infant gut microbiota and infant food allergy. Frontiers in Microbiology. 2022;13((Wang S.; Li X.; Gao Y.; Dai N.; Xing Y., yxsxz@outlook.com; Li Z., topbj163@sina.com) Department of Pediatrics, Peking University Third Hospital, Beijing, China).  408. Wang X, Li J, Liang Q, Ni X, Zhao R, Fu T, et al. Reproductive concerns and contributing factors in women of childbearing age with systemic lupus erythematosus. Clinical Rheumatology. 2022;41(8):2383-91.  409. Wright JM, Bottega N, Therrien J, Hatzakorzian R, Buithieu J, Shum-Tim D, et al. The multidisciplinary management of a mechanical mitral valve thrombosis in pregnancy: a case report and review of the literature. European Heart Journal - Case Reports. 2022;6(11).  410. Yang J, Guo Y, Dai Y. Impact of Kangaroo Mother Care Intervention on Immunological and Pulmonary Functions of Preterm Infants during Breastfeeding. Evidence-based Complementary and Alternative Medicine. 2022;2022((Yang J., yangjuan@sjzhospital.net) Department Of Children Health Care & Breast-feeding, The Fourth Hospital Of Shijiazhuang City, Shijiazhuang, China).  411. Yang ZC, Wang LX, Yu Y, Lin HY, Shih LC. Increased Risk of Postpartum Depression in Women With Allergic Rhinitis During Pregnancy: A Population-Based Case-Control Study. Annals of Otology, Rhinology and Laryngology. 2022;131(10):1137-43.  412. Yilmaz O, Kacar AS, Gogebakan E, Can C, Necef I, Mutluer T, et al. The relationship between dietary elimination and maternal psychopathology in breastfeeding mothers of infants with food allergy. Pediatric Allergy and Immunology. 2022;33(1).  413. Yonezawa K, Haruna M, Yamamoto-Hanada K, Ohya Y. Exacerbation and severity of allergic symptoms during pregnancy and their impact on mental health. International Journal of Women's Dermatology. 2022;8(1).  414. Yousif PA, Sudhakar S, Malemud C, Blumenthal DE. Cardiac Tamponade as Initial Presentation of Systemic Lupus Erythematosus in Third-Trimester Pregnancy. American Journal of Case Reports. 2022;23((Yousif P.A., patrickyousif@hotmail.com; Malemud C.; Blumenthal D.E.) Department of Medicine, Division of Rheumatology, Case Western Reserve University School of Medicine and University Hospitals Cleveland Medical Center, Cleveland, OH, United States).  415. Zaçe D, Gatta EL, Orfino A, Viteritti AM, Di Pietro ML. Knowledge, attitudes, and health status of childbearing age young women regarding preconception health - an Italian survey. Journal of Preventive Medicine and Hygiene. 2022;63(2):E270-E81.  416. Zhou Q, Tan YL, How CH, Yang LY. Breastfeeding woes: a family physician’s approach. Singapore Medical Journal. 2022;63(2):68-73.  417. Bandelow B, Allgulander C, Baldwin DS, Costa DLDC, Denys D, Dilbaz N, et al. World Federation of Societies of Biological Psychiatry (WFSBP) guidelines for treatment of anxiety, obsessive-compulsive and posttraumatic stress disorders–Version 3. Part I: Anxiety disorders. World Journal of Biological Psychiatry. 2023;24(2):79-117.  418. Banh S, Keating S. Labial artery pseudoaneurysm following preterm vaginal delivery treated with ultrasound-guided thrombin injection: A case report. Case Reports in Women's Health. 2023;39((Banh S., simon.banh@ths.tas.gov.au; Keating S.) Tasmanian Health Service - Royal Hobart Hospital, 48 Liverpool Street, Hobart, TAS, Australia).  419. Beeler PE, Stammschulte T, Dressel H. Hospitalisations Related to Adverse Drug Reactions in Switzerland in 2012–2019: Characteristics, In-Hospital Mortality, and Spontaneous Reporting Rate. Drug Safety. 2023;46(8):753-63.  420. Besag FMC, Vasey MJ. Should Antidepressants be Avoided in Pregnancy? Drug Safety. 2023;46(1):1-17.  421. Bugaeva P, Arkusha I, Bikaev R, Kamenskiy I, Pokrovskaya A, El-Taravi Y, et al. Association of breastfeeding with mental disorders in mother and child: a systematic review and meta-analysis. BMC Medicine. 2023;21(1).  422. Cui H, Mu Z. Prenatal Maternal Risk Factors Contributing to Atopic Dermatitis: A Systematic Review and Meta-Analysis of Cohort Studies. Annals of Dermatology. 2023;35(1):11-22.  423. D'Onofrio R, Omarini C, Toss A, Sperduti I, Piacentini F, Barbolini M, et al. Adjuvant Endocrine Therapy in Premenopausal Women With Hormone Receptor-Positive Early-Stage Breast Cancer: Risk Stratification in a Real-World Setting. Clinical Breast Cancer. 2023;23(7):712-20.e3.  424. Ibrahim S, Mehra R, Tantibhedhyangkul J, Bena J, Flyckt RL. Sleep and obstructive sleep apnea in women with infertility. Sleep and Breathing. 2023;27(5):1733-42.  425. Irani RA, Coscia LA, Chang E, Lappen JR. Society for Maternal-Fetal Medicine Consult Series #66: Prepregnancy evaluation and pregnancy management of patients with solid organ transplants. American Journal of Obstetrics and Gynecology. 2023;229(2):B10-B32.  426. Jenssen BP, Walley SC, Boykan R, Caldwell AL, Camenga D, Groner JA, et al. Protecting Children and Adolescents From Tobacco and Nicotine. Pediatrics. 2023;151(5).  427. Kassee C, Lunsky Y, Patrikar A, Brown HK. Impact of social-, health-, and disability-related factors on pregnancy outcomes in women with intellectual and developmental disabilities: A population-based latent class analysis. Disability and Health Journal. 2023;16(2).  428. Kinuthia J, Dettinger JC, Stern J, Ngumbau N, Ochieng B, Gómez L, et al. Risk-based versus universal PrEP delivery during pregnancy: a cluster randomized trial in Western Kenya from 2018 to 2019. Journal of the International AIDS Society. 2023;26(2).  429. Kiran BR, Vishwas GK. EPIDURAL VOLUME EXTENSION SALINE TECHNIQUE FOR ANESTHETIC MANAGEMENT OF PERIPARTUM CARDIOMYOPATHY CASES FOR EMERGENCY CAESAREAN SECTION- A CASE SERIES. International Journal of Pharmaceutical Sciences and Research. 2023;14(4):1851-4.  430. Korğalı EÜ, Tunç G. The levels of postpartum depression, anxiety, and hopelessness of the mothers of infants receiving therapeutic hypothermia in NICU. Children's Health Care. 2023;52(4):469-86.  431. Lee MH, Wu MC, Wang YH, Wei JCC. Maternal constipation is associated with allergic rhinitis in the offspring: A nationwide retrospective cohort study. PLoS ONE. 2023;18(10 October).  432. Leslie K, Barker LC, Brown HK, Chen S, Lee Dennis C, Ray JG, et al. Risk of interpersonal violence during and after pregnancy among people with schizophrenia: a population-based cohort study. CMAJ Canadian Medical Association Journal. 2023;195(9):E322-E9.  433. Li Y, Xie T, Cardoso Melo RD, de Vries M, Lakerveld J, Zijlema W, et al. Longitudinal effects of environmental noise and air pollution exposure on autism spectrum disorder and attention-deficit/hyperactivity disorder during adolescence and early adulthood: The TRAILS study. Environmental Research. 2023;227((Li Y., y.li@umcg.nl; Xie T., t.xie@umcg.nl; Cardoso Melo R.D.; Hartman C.A.) University of Groningen, University Medical Center Groningen, Interdisciplinary Center Psychopathology and Emotion Regulation, Department of Psychiatry, Groningen, Netherlands).  434. Liu YC, Liao YT, Chen VCH, Chen YL. Association Between Maternal Mood Disorders and Schizophrenia and the Risk of Type 1 Diabetes in Offspring: A Nationwide Cohort Study. Neuropsychiatric Disease and Treatment. 2023;19((Liu Y.-C.) Department of Psychiatry, Changhua Christian Children’s Hospital, Changhua, Taiwan):2511-8.  435. Majdinasab E, Datta P, Krutsch K, Baker T, Hale TW. Pharmacokinetics of Ketamine Transfer into Human Milk. Journal of Clinical Psychopharmacology. 2023;43(5):407-10.  436. Mandima P, Baltrusaitis K, Montepiedra G, Aaron L, Mathad J, Onyango-Makumbi C, et al. Prevalence of neurotoxicity symptoms among postpartum women on isoniazid preventive therapy and efavirenz-based treatment for HIV: an exploratory objective of the IMPAACT P1078 randomized trial. BMC Pregnancy and Childbirth. 2023;23(1).  437. Mehl SC, Peiffer S, Powell P, Belfort MA, Lee TC, Keswani SG, et al. Association of Socioeconomic Status with Empowerment, Depression, and Anxiety in Pregnancies Complicated by Fetal Surgical Anomalies. Journal of Pediatric Surgery. 2023;58(6):1111-5.  438. Nelson A, Lebelo K, Cassidy T, Duran LT, Mantangana N, Mdani L, et al. Postnatal clubs: Implementation of a differentiated and integrated model of care for mothers living with HIV and their HIV-exposed uninfected babies in Cape Town, South Africa. PLoS ONE. 2023;18(11 November).  439. Ngure K, Friedland BA, Szydlo DW, Roberts ST, Garcia M, Levy L, et al. Baseline preferences for oral pre-exposure prophylaxis (PrEP) or dapivirine intravaginal ring for HIV prevention among adolescent girls and young women in South Africa, Uganda and Zimbabwe (MTN-034/IPM-045 study). PLoS ONE. 2023;18(6 June).  440. Nguyen RHN, Knapp EA, Li X, Camargo CA, Conradt E, Cowell W, et al. Characteristics of Individuals in the United States Who Used Opioids during Pregnancy. Journal of Women's Health. 2023;32(2):161-70.  441. OjiNjideka Hemphill N, Pezley L, Steffen A, Elam G, Kominiarek MA, Odoms-Young A, et al. Feasibility Study of Lactobacillus Plantarum 299v Probiotic Supplementation in an Urban Academic Facility among Diverse Pregnant Individuals. Nutrients. 2023;15(4).  442. Peiffer S, Mehl SC, Powell P, Haltom TM, Lee TC, Keswani SG, et al. Maternal Anxiety and Empowerment in Pregnancies Complicated By Fetal Surgical Anomalies: A Mixed Methods Study. Journal of Surgical Research. 2023;292((Peiffer S.; Mehl S.C.; Lee T.C.; Keswani S.G.; King A., axking3@texaschildrens.org) Michael E. DeBakey Department of Surgery, Baylor College of Medicine, Houston, TX, United States):22-9.  443. Przulj D, Pesola F, Smith KM, McRobbie H, Coleman T, Lewis S, et al. Helping pregnant smokers quit: a multicentre randomised controlled trial of electronic cigarettes versus nicotine replacement therapy. Health Technology Assessment. 2023;27(13):1-53.  444. Rojop N, Calvimontes DM, Barrios E, Lamb MM, Paniagua-Avila A, Monzon J, et al. COVID-19 Attitudes and Vaccine Hesitancy among an Agricultural Community in Southwest Guatemala: A Cross-Sectional Survey. Vaccines. 2023;11(6).  445. Shirwani A, Kuller JA, Dotters-Katz SK, Addae-Konadu K. Nicotine Use During Pregnancy: Cessation and Treatment Strategies. Obstetrical and Gynecological Survey. 2023;78(10):589-97.  446. Smew AI, Lundholm C, Gong T, Sävendahl L, Lichtenstein P, Brew BK, et al. Maternal depression or anxiety during pregnancy and offspring type 1 diabetes: A population-based family-design cohort study. BMJ Open Diabetes Research and Care. 2023;11(2).  447. Sterba KR, Johnson EE, Douglas E, Aujla R, Boyars L, Kruis R, et al. Implementation of a women’s reproductive behavioral health telemedicine program: a qualitative study of barriers and facilitators in obstetric and pediatric clinics. BMC Pregnancy and Childbirth. 2023;23(1).  448. Sun L, Tang M, Peng M, Xu P, Wang Y. Ritodrine-induced rhabdomyolysis and psychiatric symptoms: a case report and literature review. BMC Pregnancy and Childbirth. 2023;23(1).  449. Vijayashankar SS, Sanatani G, Franciosi S, Moodley S, Ting JY. Left ventricular dysfunction in the immediate post-natal period. Translational Pediatrics. 2023;12(1):13-9.  450. Wang Z, Sun H, Zhang C, Lu L, Zhang L, Wang D. Outcomes of acute type A aortic dissection repair during pregnancy. International Journal of Gynecology and Obstetrics. 2023;161(3):927-33.  451. Weld J, Lee B, Loomba RS, Siddiqui S, Jaji A, Vricella L, et al. Tricuspid atresia and common arterial trunk: a rare form of CHD. Cardiology in the Young. 2023;33(7):1192-5.  452. Xu YQ, Gou Y, Yuan JJ, Zhu YX, Ma XM, Chen C, et al. Peripheral Blood Inflammatory Cytokine Factors Expressions are Associated with Response to Acupuncture Therapy in Postpartum Depression Patients. Journal of Inflammation Research. 2023;16((Xu Y.-Q.; Gou Y.; Yuan J.-J.; Zhu Y.-X.; Ma X.-M.; Huang X.-X.; Yang Z.-X., 001188@gzucm.edu.cn; Zhou Y.-M., zym_star@163.com) The Fourth Clinical Medical College of Guangzhou University of Chinese Medicine, Shenzhen Traditional Chinese Medicine Hospital, Guangdong Province, Shenzhen, China):5189-203.  453. Yu S, Zhang Y, Chen Z, Song J, Wang C. A Novel Compound Heterozygous Gene Mutation of Dolichol Kinase Deficiency (DOLK-CDG). Endocrine, Metabolic and Immune Disorders - Drug Targets. 2023;23(2):235-41.  454. Zamaniyan M, Rahmani Z, Ghasemian R, Karimi Z, Arab RK, Ebadi A, et al. Maternal and Neonatal Outcomes in Pregnant Women With COVID-19 Admitted to Imam Khomeini Hospital of Sari, Iran, 2019-2020. Archives of Clinical Infectious Diseases. 2023;18(1).  455. Zhou JX, Guo Y, Teng YZ, Zhu LL, Lu J, Hao XM, et al. Maternal anxiety during pregnancy and children's asthma in preschool age: The Ma'anshan birth cohort study. Journal of Affective Disorders. 2023;340((Zhou J.-X.; Guo Y.; Teng Y.-Z.; Zhu L.-L.; Lu J.; Hao X.-M.; Yan S.-Q.; Tao F.-B.; Huang K., ahmuhuangk@163.com) Department of Maternal, Child and Adolescent Health, School of Public Health, Anhui Medical University, China):312-20.  456. Zhuk A, Tsylko A, Nazarava V, Migun A. Extracorporeal membrane oxygenation in 34 days old infant with SARS-COV-2 associated pneumonia. Perfusion (United Kingdom). 2024;39(5):1014-6. |
| **Studies excluded in full-text review with reasons (n = 19)** | |
| Reasons | Citations |
| Maternal depression not evaluated as exposure | 1. Continued exposure to maternal distress in early life is associated with an increased risk of childhood asthma. Am J Respir Crit Care Med. 2008;177(2):142-7. |
| Maternal depression not evaluated as exposure | 2. Mothers' anxiety during pregnancy is associated with asthma in their children. J Allergy Clin Immunol. 2009;123(4):847-53.e11. |
| Outcome of interest not reported | 3. Maternal depression related to infant's wheezing. Pediatr Allergy Immunol. 2011;22(6):608-13. |
| Data not available | 4. Child asthma and change in elevated depressive symptoms among mothers of children of a birth cohort from Quebec. Women Health. 2011;51(5):461-81. |
| Outcome of interest not reported | 5. Child allergic symptoms and mental well-being: the role of maternal anxiety and depression. J Pediatr. 2014;165(3):592-9.e5. |
| Maternal depressive symptoms not evaluated in perinatal periods | 6. Maternal depressive symptoms across early childhood and asthma in school children: findings from a Longitudinal Australian Population Based Study. PLoS One. 2015;10(3):e0121459. |
| Outcome of interest not reported | 7. Postpartum depression, a direct and mediating risk factor for preschool wheeze in girls. Pediatr Pulmonol. 2016;51(4):349-57. |
| Maternal depressive symptoms not evaluated in perinatal periods | 8. Maternal depressive symptoms, maternal asthma, and asthma in school-aged children. Ann Allergy Asthma Immunol. 2017;118(1):55-60.e1. |
| Maternal depressive symptoms not evaluated in perinatal periods | 9. Relationships among Maternal Stress and Depression, Type 2 Responses, and Recurrent Wheezing at Age 3 Years in Low-Income Urban Families. Am J Respir Crit Care Med. 2017;195(5):674-681. |
| Maternal depressive symptoms evaluated as a continuous variable | 10. Early-life home environment and risk of asthma among inner-city children. J Allergy Clin Immunol. 2018;141(4):1468-1475. |
| Outcome of interest not reported | 11. Associations between maternal mental health and early child wheezing in a South African birth cohort. Pediatr Pulmonol. 2018;53(6):741-754. |
| Outcome of interest not reported | 12. Longitudinal Phenotypes of Respiratory Health in a High-Risk Urban Birth Cohort. Am J Respir Crit Care Med. 2019;199(1):71-82. |
| Maternal depression not evaluated as exposure | 13. The influence of prenatal mental health service use on the incidence of childhood asthma: a population-based cohort study. J Asthma. 2019;56(4):395-403. |
| Maternal depression not evaluated as exposure | 14. Maternal Distress During Pregnancy and Recurrence in Early Childhood Predicts Atopic Dermatitis and Asthma in Childhood. Chest. 2020;158(1):57-67 |
| Outcome of interest not reported | 15. Childhood Allergies: The Role of Maternal Depression and Anxiety, and Family Strain. Children (Basel). 2021;8(3):185. |
| Outcome of interest not reported | 16. A model of perinatal stress and childhood wheezing: ELSPAC-CZ cohort. Pediatr Pulmonol. 2021;56(6):1471-1483. |
| Outcome of interest not reported | 17. Maternal prenatal psychological distress associates with offspring early-life wheezing - FinnBrain Birth Cohort. Pediatr Allergy Immunol. 2022;33(1):e13706. |
| Maternal depression not evaluated as exposure | 18. Maternal mental illness and child atopy: a UK population-based, primary care cohort study. Br J Gen Pract. 2023;73(737):e924-e931 |
| Maternal depression not evaluated as exposure | 19.0Maternal mental health disorders and offspring asthma and allergic diseases: The role of child mental health. Pediatr Allergy Immunol. 2024;35(2):e14085. |
| **Studies included in the meta-analysis (n = 10)** | |
| Included | Citations |
|  | 1. Guxens M, Sonnenschein-van der Voort AM, Tiemeier H, Hofman A, Sunyer J, de Jongste JC, et al. Parental psychological distress during pregnancy and wheezing in preschool children: the Generation R Study. J Allergy Clin Immunol. 2014;133(1):59-67 e1-12.  2. Liu X, Olsen J, Pedersen LH, Agerbo E, Yuan W, Li J. Antidepressant use during pregnancy and asthma in the offspring. Pediatrics. 2015;135(4):e911-7.  3. Wen HJ, Chiang TL, Lin SJ, Guo YL. Predicting risk for childhood asthma by pre-pregnancy, perinatal, and postnatal factors. Pediatr Allergy Immunol. 2015;26(3):272-9.  4. Tomfohr LM, Bayrampour H, Tough S. Maternal History of Childhood Abuse and Risk of Asthma and Allergy in 2-Year-Old Children. Psychosom Med. 2016;78(9):1031-42.  5. Kozyrskyj AL, Letourneau NL, Kang LJ, Salmani M. Associations between postpartum depressive symptoms and childhood asthma diminish with child age. Clin Exp Allergy. 2017;47(3):324-30.  6. Zhou C, Ibanez G, Miramont V, Steinecker M, Baiz N, Banerjee S, et al. Prenatal maternal depression related to allergic rhinoconjunctivitis in the first 5 years of life in children of the EDEN mother-child cohort study. Allergy Rhinol (Providence). 2017;8(3):132-8.  7. Magnus MC, Wright RJ, Roysamb E, Parr CL, Karlstad O, Page CM, et al. Association of Maternal Psychosocial Stress With Increased Risk of Asthma Development in Offspring. Am J Epidemiol. 2018;187(6):1199-209.  8. R van Meel E, Saharan G, Jaddoe VW, de Jongste JC, Reiss IK, Tiemeier H, et al. Parental psychological distress during pregnancy and the risk of childhood lower lung function and asthma: a population-based prospective cohort study. Thorax. 2020;75(12):1074-81.  9. Alcala CS, Orozco Scott P, Tamayo-Ortiz M, Hernandez Chavez MDC, Schnaas L, Carroll KN, et al. Longitudinal assessment of maternal depression and early childhood asthma and wheeze: Effect modification by child sex. Pediatr Pulmonol. 2023;58(1):98-106.  10. Shi YY, Wei Q, Ma X, Zhang Y, Wang L, Shi HJ. Maternal affective and stress-related factors during pregnancy affect the occurrence of childhood allergic diseases: A Shanghai MCPC study. J Psychosom Res. 2023;165:111142. |
